# Pathogen-based target attainment of optimized continuous infusion dosing regimens of piperacillin-tazobactam and meropenem in surgical ICU patients: a prospective single center observational study

**DOI:** 10.1186/s13613-023-01129-6

**Published:** 2023-04-29

**Authors:** Thomas De Corte, Jarne Verhaeghe, Sofie Dhaese, Sarah Van Vooren, Jerina Boelens, Alain G. Verstraete, Veronique Stove, Femke Ongenae, Liesbet De Bus, Pieter Depuydt, Sofie Van Hoecke, Jan J. De Waele

**Affiliations:** 1 Department of Internal Medicine and Pediatrics, Faculty of Medicine and Health Sciences, Ghent University, Ghent, Belgium; 2 Department of Intensive Care Medicine, Ghent University Hospital, Ghent, Belgium; 3 IDLab, Ghent University–Imec, Ghent, Belgium; 4 Department of Diagnostic Sciences, Faculty of Medicine and Health Sciences, Ghent University, Ghent, Belgium; 5 Department of Laboratory Medicine, Ghent University Hospital, Ghent, Belgium

**Keywords:** Piperacillin-tazobactam, Meropenem, Therapeutic drug monitoring, Continuous infusion

## Abstract

**Background:**

Several studies have indicated that commonly used piperacillin-tazobactam (TZP) and meropenem (MEM) dosing regimens lead to suboptimal plasma concentrations for a range of pharmacokinetic/pharmacodynamic (PK/PD) targets in intensive care unit (ICU) patients. These targets are often based on a hypothetical worst-case scenario, possibly overestimating the percentage of suboptimal concentrations. We aimed to evaluate the pathogen-based clinically relevant target attainment (CRTA) and therapeutic range attainment (TRA) of optimized continuous infusion dosing regimens of TZP and MEM in surgical ICU patients.

**Methods:**

A single center prospective observational study was conducted between March 2016 and April 2019. Free plasma concentrations were calculated by correcting total plasma concentrations, determined on remnants of blood gas samples by ultra-performance liquid chromatography with tandem mass spectrometry, for their protein binding. Break points (BP) of identified pathogens were derived from epidemiological cut-off values. CRTA was defined as a corrected measured total serum concentration above the BP and calculated for increasing BP multiplications up to 6 × BP. The upper limit of the therapeutic range was set at 157.2 mg/L for TZP and 45 mg/L for MEM. As a worst-case scenario, a BP of 16 mg/L for TZP and 2 mg/L for MEM was used.

**Results:**

781 unique patients were included with 1036 distinctive beta-lactam antimicrobial prescriptions (731 TZP, 305 MEM) for 1003 unique infections/prophylactic regimens (750 TZP, 323 MEM). 2810 samples were available (1892 TZP, 918 MEM). The median corrected plasma concentration for TZP was 86.4 mg/L [IQR 56.2–148] and 16.2 mg/L [10.2–25.5] for MEM. CRTA and TRA was consistently higher for the pathogen-based scenario than for the worst-case scenario, but nonetheless, a substantial proportion of samples did not attain commonly used PK/PD targets.

**Conclusion:**

Despite these pathogen-based data demonstrating that CRTA and TRA is higher than in the often-used theoretical worst-case scenario, a substantial proportion of samples did not attain commonly used PK/PD targets when using optimised continuous infusion dosing regimens. Therefore, more dosing optimization research seems warranted. At the same time, a ‘pathogen-based analysis’ approach might prove to be more sensible than a worst-case scenario approach when evaluating target attainment and linked clinical outcomes.

**Supplementary Information:**

The online version contains supplementary material available at 10.1186/s13613-023-01129-6.

## Introduction

Up to 66% of patients admitted to the intensive care unit (ICU) will be treated with an antimicrobial at some point during admission [1]. Piperacillin-tazobactam (TZP) and meropenem (MEM), two broad spectrum beta-lactam antimicrobials, are among the most frequently prescribed antimicrobials in ICUs worldwide [2]. As knowledge about their use evolved, dosing regimens based on covariates, such as the renal function of the patient, have been developed to better suit their use in the ICU population. Despite this progress, some studies still indicate that a large proportion of patients is sub optimally treated when considering relevant pharmacokinetic/pharmacodynamic (PK/PD) targets [3]. Recently, a joint statement by the European Society of Intensive Care Medicine (ESICM), the Pharmacokinetic/Pharmacodynamic and Critically Ill Patient Study Groups of the European Society of Clinical Microbiology and Infectious Diseases (ESCMID), the International Association for Therapeutic Drug Monitoring and Clinical Toxicology (IATDMCT) and the International Society of Antimicrobial Chemotherapy (ISAC) was published, advocating the use of therapeutic drug monitoring (TDM) to optimize beta-lactam antimicrobial treatment for the individual ICU patient [4]. To date, the use of beta-lactam TDM is not yet common practice for ICU clinicians as the effect of TDM of these antimicrobials on morbidity and mortality still has to be proven, and the technology for TDM is not widely available [5, 6]. However, it is plausible that the proportion of patients not attaining the desired target concentrations in these TDM studies might be overestimated, as typically a hypothetical worst-case scenario is used when no pathogen is identified or during empirical therapy. In these instances, researchers typically use the epidemiological breakpoint (BP) of *Pseudomonas aeruginosa* (16 mg/L TZP – 2 mg/L MEM). Many infections, however, are caused by pathogens that have a lower BP than *Pseudomonas aeruginosa*, which could influence the study results.

We, therefore, aimed to describe measured plasma concentrations of TZP and MEM attained by optimized continuous infusion dosing regimens, as well as to describe the respective patient and infection characteristics in a large surgical ICU study cohort. We determined the difference on clinically relevant target attainment (CRTA) and therapeutic range attainment (TRA) between a hypothetical, worst-case scenario and a pathogen-based scenario in this population. Additionally, we aimed to describe the effect of kidney function, BP and choice of PK/PD target on target attainment in this study population.

## Methods

### Study population

Between March 2016 and April 2019, patients admitted to the surgical ICU of Ghent University hospital, receiving either piperacillin-tazobactam (4 g/0.5 g powder for solution for infusion; Fresenius Kabi n.v., Schelle, Belgium) or meropenem (0.5 g or 1 g powder for solution for infusion; Fresenius Kabi n.v., Schelle, Belgium) in continuous infusion, in need of routine blood sampling, and above the age of 18 years old were included. Initial dosing regimens and any subsequent dosing modifications were based on creatinine clearance, calculated from creatinine measurements on a once-daily 8-h urinary collection and a plasma sample. When no urine was available, the creatinine clearance calculated by means of the Chronic Kidney Disease Epidemiology Collaboration formula (CKD-EPI) was used. Dosing regimens are provided in Table 1. Measured antimicrobial plasma concentrations were not disclosed to the treating physicians, hence no dosing adaptations were performed based on the measured concentrations.

**Table 1:** Dosing regimens for piperacillin-tazobactam and meropenem according to renal function

	Loading dose	Continuous infusion
Piperacillin-tazobactam		
Creatinine clearance > 30 mL/min	4/0.5 g given over 30 min	16/2 g every 24 h
Creatinine clearance 15–29 mL/min		12/1.5 g every 24 h
Creatinine clearance < 15 mL/min		8/1 g every 24 h
Intermittent RRT		8/1 g every 24 h + repeat loading dose within 2 h after every RRT session
Continuous RRT		12/1.5 g every 24 h
Meropenem		
Creatinine clearance > 30 mL/min	1 g given over 30 min	3 g every 24 h
Creatinine clearance 15–29 mL/min		2 g every 24 h
Creatinine clearance < 15 mL/min		1 g every 24 h
Intermittent RRT		1 g every 24 h + repeat loading dose within 2 h after every RRT session
Continuous RRT		3 g every 24 h
Meropenem high dose	2 g given over 30 min	6 g every 24 h

### Samples and sample analysis

Remnants of the RAPIDLyte Arterial Blood syringes (Siemens) taken as part of the routine blood sampling every morning at 6 a.m. were used as study material. Samples were stored at 4°C for maximally 3h, centrifuged and the supernatant was frozen at −80°C within 1h awaiting batch analysis. Total plasma concentrations of TZP/MEM were analyzed by the Department of Laboratory Medicine of Ghent University Hospital using a validated fast ultra-performance liquid chromatographic method with tandem mass spectrometric detection (UPLC-MS/MS) [7]. A 15 µL sample was mixed with 100 µL internal standard solution (3 µg/ml deuterated internal standards in acetonitrile) and vortexed for 3 min. at 1400 rounds/min. After centrifugation for 5 min. at 16,000 g, 100 µL of the supernatant was transferred into an autosampler vial which contained 400 µL of milli-Q water and vortexed for 3 min. The UPLC–MS/MS system consisted of a Waters Acquity UPLC instrument coupled to a TQD triple-quadrupole mass spectrometer (Waters Corp., Milford, MA). Separations were performed on an Acquity UPLC BEH C18 column (100 mm × 2.1 mm, 1.7 µm particle size) equipped with a 0.2 µm precolumn filter unit and a guard column (Waters Corp., Milford, MA). Analytes were measured in the multiple reaction monitoring (MRM) mode. The flow rate was set at 0.4 mL/min. The column and autosampler tray temperature was set at 50°C and 4°C, respectively. Forty µL of the extract was injected into the column. The MS/MS instrument was operated with a capillary voltage of 1.00 kV, a source temperature of 140 °C and desolvation gas (nitrogen) at 400 °C with a flow of 800 L/h. Analytes were measured in the positive electrospray ionization (ESI +) mode. The deuterated standards piperacillin-D_5_ and meropenem-D_6_ from Toronto Research Chemicals (Ontario, Canada) were used as internal standards. Data were acquired using Masslynx 4.1 software and processed using Quanlynx 4.1 software (Waters Corp., Milford, MA). For TZP, a protein binding of 30% was assumed, for which the measured concentration was corrected [8]. For MEM, the influence of protein binding was considered to be negligible [9]. Samples taken within a time frame of 12h after start of therapy or a dose change were considered as non-steady state samples, while the other samples were considered to be in a steady state.

### Data collection

All patient data were prospectively collected during their stay and extracted from the Intensive Care Information System database (Centricity Critical Care®, GE Healthcare, Machelen, Belgium). Extracted data included, among others, patient demographics, severity scores, renal function on the day of sampling, and lab results. Data regarding the reason for antimicrobial prescription as well as associated microbiological data were extracted from the Computer-based Surveillance and Alerting of nosocomial infections, Antimicrobial Resistance and Antibiotic consumption in the ICU (COSARA) database [10]. In this database, all prescribed antimicrobials are linked with a suspected focus of infection and a pathogen (if available). Chronic kidney dysfunction (CKD) and acute kidney injury (AKI) definitions provided by the “Kidney Disease, Improving Global Outcomes” were used [11, 12]. Augmented renal clearance (ARC) was defined as a measured creatinine clearance on an 8-h urinary collection > 130 ml/min [13]. Kidney function at the time of sampling was categorized as “ARC”, “Stable kidney function”, “AKI I”, “AKI II”, “AKI III” or “RRT”. Samples for which the kidney function was not evaluable at the time of sampling were excluded.

### Break points, therapeutic range and scenarios

As minimal inhibitory concentrations are not routinely determined in our microbiology laboratory, published clinical breakpoints (BP) from The European Committee on Antimicrobial Susceptibility Testing (EUCAST) 2022 and Société Française de Microbiologie (SFM) 2022 were used [14, 15]. In case both organizations reported BPs, the EUCAST BP was preferred. In case of a polymicrobial infection, the BP from the pathogen with the highest BP was retained. Clinically relevant target attainment (CRTA) was defined as a measured total plasma concentration, corrected for protein binding, above the BP. CRTA was evaluated for increasing multiplications of the initial BP with a maximum of 6 × BP. As an upper limit of the therapeutic range, an unbound concentration of 157.2 mg/L was chosen for TZP and 45 mg/L for MEM [16, 17].

For the pathogen-based scenario, all samples for which a BP was determined as described above were used. For the worst-case scenario, all samples for which a BP was determined were assigned a worst-case BP of 16 mg/L for TZP and 2 mg/L for MEM. Hence, for the TZP pathogen-based scenario, the lower boundary was variable and determined by the BP multiplication while the upper limit was set at 157.2 mg/L. The therapeutic range for the worst-case TZP scenario was also variable and determined by multiplying 16 mg/L with the evaluated BP multiplication, while the upper limit remained fixed at 157.2 mg/L.

### Ethical approval and statistical analysis

This study was performed in accordance with the Declaration of Helsinki. Ethical approval was obtained from the Ghent University Hospital Ethics Committee (Registration Number 2016/0264). Informed consent was obtained for all participants via opting out before participation.

All statistical analyses were performed using R studio (v 4.2.1) [18]. Continuous variables are presented as mean and standard deviation for normally distributed data and as median and interquartile range for non-normally distributed data. Categorical variables are presented as counts and percentages of total evaluable instances (given between brackets) unless explicitly stated otherwise. Distribution of continuous variables was evaluated by means of a Q–Q plot and Shapiro–Wilk test. Comparison of non-normally distributed continuous variables with categorical variables was performed by means of the Kruskal–Wallis test. A *p*-value of < 0.05 was considered statistically significant. All samples were used for the description of the study population, the identified infection focus and pathogens, the distribution of measured concentrations according to the renal function, and the evaluation of toxicity. Only samples for which a pathogen was identified as described above, were used for CRTA and TRA evaluation.

## Results

### Study population

A total of 781 unique patients were included for a total of 878 unique ICU admissions (704 TZP, 275 MEM). A total of 97 patients received both TZP and MEM during the same ICU stay. Treatment with TZP was started on a median of 0 [0–4] days after ICU admission, while this was 1 [0–10] for MEM. The median time to sampling following the start of antimicrobials in the ICU was 2 days [1–5] for TZP and 4 days [2–7] for MEM. A total of 639 samples were taken within a time frame of 12 h after a dose change or ICU admission and hence were considered to be non-steady state samples (430 TZP, 209 MEM). 1036 distinctive beta-lactam antimicrobial prescriptions were initiated (731 TZP, 305 MEM) for 1003 unique infections/prophylactic regimens (750 TZP, 323 MEM). 2810 samples were available (1892 TZP, 918 MEM), with a median of 2 TZP [1–3] as well as 2 MEM [1–5] samples per unique TZP or MEM treatment. A full overview of the characteristics per unique admission can be found in Table 2.

**Table 2:** Admission baseline characteristics

	Piperacillin-tazobactam (*N* = 704)	Meropenem (*N* = 275)	Admissions where both MEM and TZP were used (*N* = 97)
Demographics			
Age (years)	62.5 [48.0–72.0]	65 [51.0–73.0]	62 [49.0–72.0]
Gender (% male)	63.9	67.6	67
BMI	24.97 [22.59–28.4] *(699)*	26.12 [23.46–29.41] *(273)*	26.21 [23.7–29.04]
Race			
Caucasian	96.4%	96.4% *(274)*	97.0%
Black	0.7%	0.4% *(274)*	0.0%
Asian	0.6%	0.4% *(274)*	0.0%
Other	1.3%	1.4% *(274)*	3.0%
Not specified	1.0%	1.4% *(274)*	0.0%
Comorbidities			
Baseline creatinine (mg/dL)	0.83 [0.68–1.15] *(599)*	0.84 [0.66–1.2] *(229)*	0.84 [0.68–1.16]
Baseline kidney function			
Unknown	14.91%	16.73%	0%
KDIGO G1	41.34%	37.45%	39.18%
KDIGO G2	27.30%	23.64%	20.62%
KDIGO G3a	9.09%	7.64%	8.25%
KDIGO G3b	5.40%	6.55%	6.19%
KDIGO G4	2.56%	4.36%	3.09%
KDIGO G5	1.14%	1.45%	2.06%
RRT	2.27%	2.18%	1.03%
SOFA on admission	7.0 [4.0–11.0] (*697*)	8.0 [4.0–12.0] (*270*)	10.0 [5.0–13.0]
Apache 4 score on admission	106 [79.0–134]	118 [88.0–148.0]	118 [88.–152.0]
Reason for ICU admission			
Monitoring or post op management	36.6%	28.4%	22.7%
Respiratory failure	13.9%	17.5%	16.5%
Septic shock	12.2%	18.5%	17.5%
Hypovolemic/hemorrhagic shock	7.5%	5.8%	8.2%
Severe sepsis	6.4%	10.5%	9.2%
Other	23.4%	19.3%	25.9%
Surgery as part of reason for admission			
No surgery	43.8%	58.5%	55.7%
Planned surgery	28.6%	19.6%	18.6%
Emergency surgery	27.5%	21.8%	25.8%
Disease severity			
SOFA on starting day AB	8.0 [5.0–11.0] (*702*)	8.0 [4.0–12.0] (*270*)	10 [6.0–10.0]
Outcomes			
ICU length of Stay	7.0 [3.0–17.0]	13.0 [6.0–25.0]	23.79 [14.0–33.1]
ICU mortality	15.20%	20.70%	20.60%

All continuous variables are presented as median with IQR [Q1–Q3]. Categorical variables are presented as percentage of total evaluable instances [given as (*N* =) in the column header]. If less evaluable instances were available, the total number of available instances is given between ()

*BMI* Body mass index,* KDIGO *Kidney disease, improving global outcomes stage*, RRT* Renal replacement therapy*, SOFA* Sequential organ failure assessment

### Pathogens identified, infection focus and sample characteristics

In 37.5% of infections, no pathogen could be identified; 26.0% of infections were polymicrobial. A breakdown of the distribution of infection foci and the 10 most frequently identified pathogens as a percentage of total identified pathogens can be found in Additional file 1: Tables S1 and S2. Distributions of number of measured samples according to BP of the pathogen and renal function can be found in Table 3. Characteristics of the samples taken are listed in Additional file 1: Table S3. For 1671 TZP and 834 MEM samples, evaluation of physician adherence to the described dosing guidelines was possible. For 83.54% of evaluable TZP and 90.41% of evaluable MEM samples, the physicians adhered to the guidelines (Additional file 1: Table S4). An Evaluation of dosing appropriateness according to AKI stage on the sampling day can be found in Additional file 1: Table S5.

**Table 3:** Distribution of samples according to BP and kidney function [counts and *(*percentages*)]*

	Piperacillin-tazobactam				Meropenem	
	0.25 mg/L	8 mg/L	16 mg/L	BP unknown	2 mg/L	BP unknown
	(*N* = 10)	(*N* = 808)	(*N* = 257)	(*N* = 817)	(*N* = 631)	(*N* = 297)
ARC	4 *(40.0)*	168 *(20.8)*	59 *(23.0)*	149 *(18.2)*	126 *(20.3)*	52 *(17.5)*
Stable kidney function	4 *(40.0)*	335 *(41.5)*	115 *(44.8)*	354 *(43.3)*	274 *(44.1)*	139 *(46.8)*
AKI I	2 *(20.0)*	110 *(13.6)*	32 *(12.5)*	90 (11.0)	63 *(10.1)*	34 *(11.5)*
AKI II	0 *(0.0)*	70 *(8.6)*	25 *(9.7)*	93 (11.4)	41 *(6.6)*	29 *(9.8)*
AKI III	0 *(0.0)*	33 *(4.1)*	1 *(0.4)*	29 (3.6)	17 *(2.7)*	7 *(2.4)*
RRT	0 *(0.0)*	92 *(11.4)*	25 *(9.7)*	102 (12.5)	100 *(16.1)*	36 *(12.1)*

*BP* Break point*, ARC* Augmented renal clearance*, AKI* Acute kidney injury*, RRT* Renal replacement therapy

### Antibiotic concentrations

#### Piperacillin-tazobactam

**Fig. 1:**
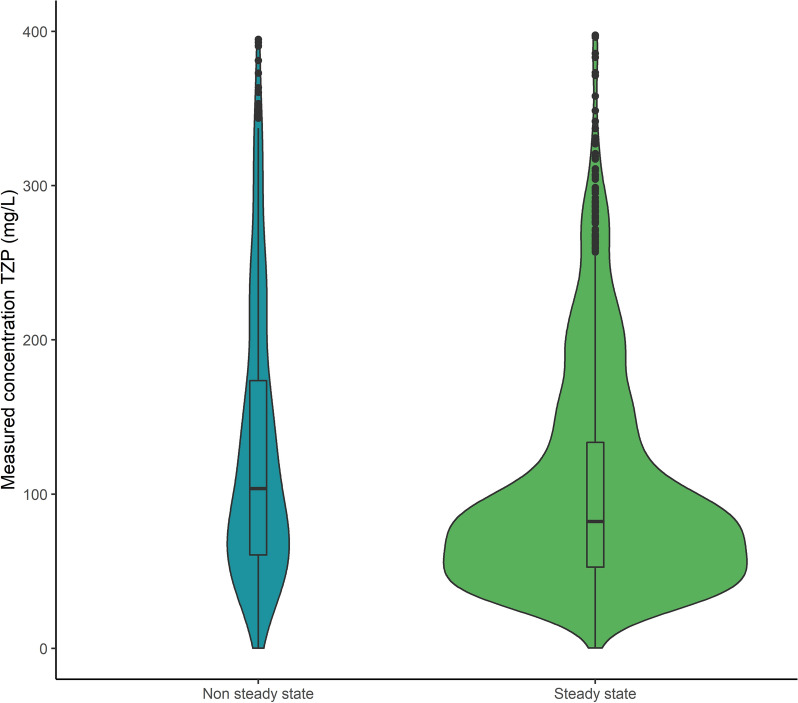
Violin plot of free TZP concentrations according to sample state (*p* < 0.01)

**Fig. 2:**
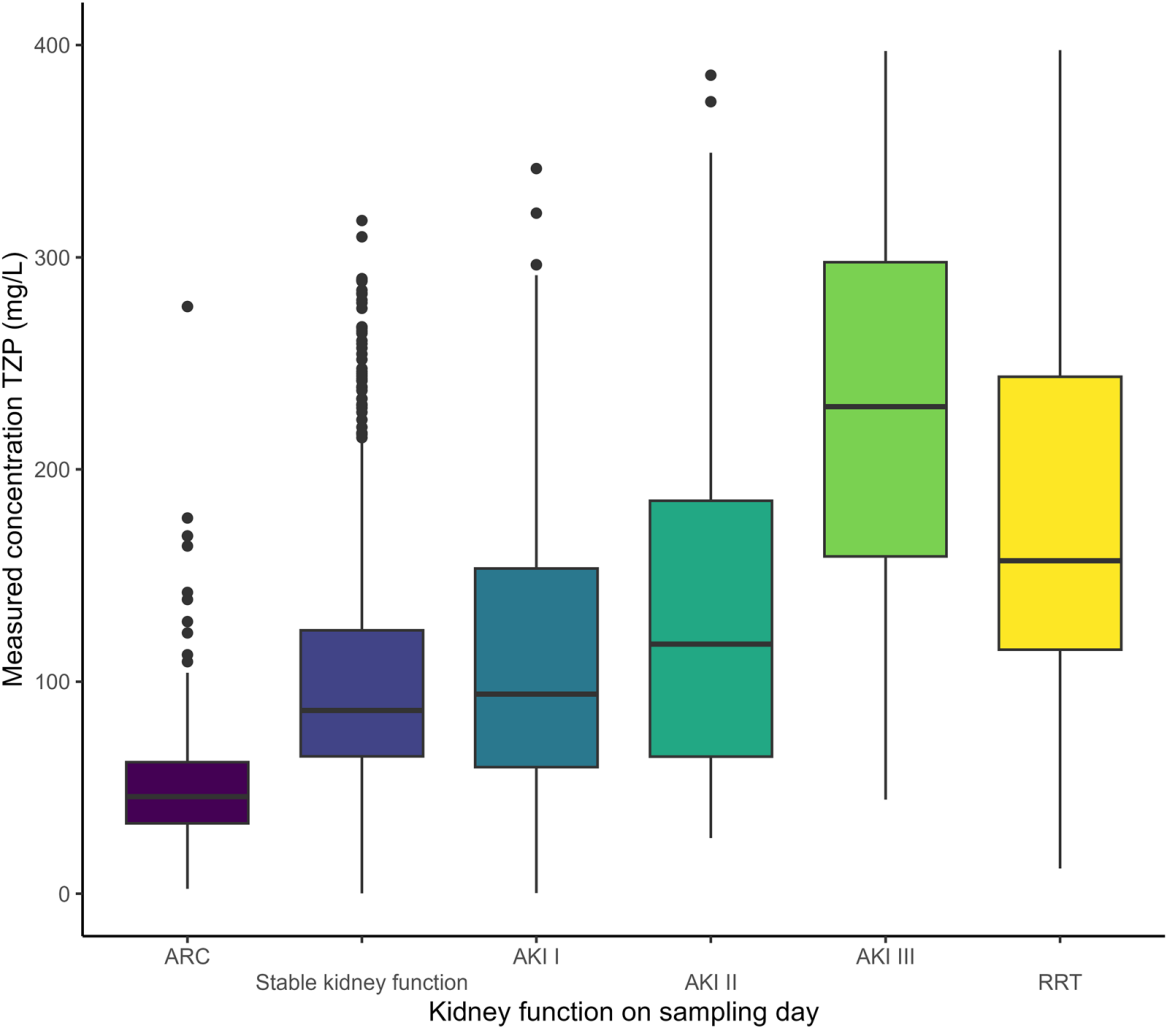
Boxplot of free TZP concentrations according to renal function on the sampling day

The median measured concentration for TZP was 106 mg/L [64.2–182] for non-steady state samples and 83.2 mg/L [54.1–135] for steady state samples (Fig. 1— *p* < 0.01). Distributions of measured concentrations according to the renal function are shown in Fig. 2. The lowest concentrations were measured when ARC was present on the day of sampling, while increasingly higher concentrations with a widening IQR-range were measured with increasing stages of AKI. Measured concentrations for RRT samples were also higher than when the renal function was stable.

**Fig. 3:**
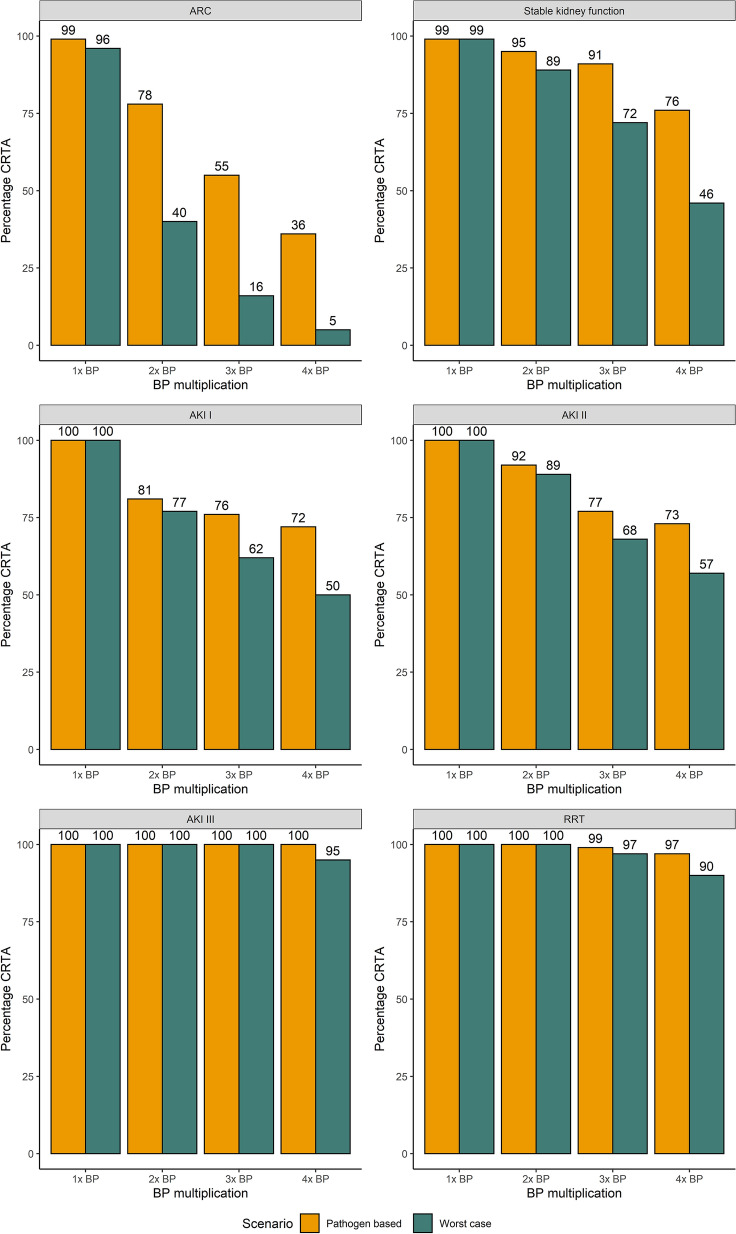
Percentage of CRTA for all TZP samples according to renal function and BP multiplication targeted

Figure 3 compares clinically relevant target attainment between the pathogen-based and the worst-case scenario according to the renal function on the day of sampling and increasing BP multiplication (up to 4 × BP) as a target. CRTA decreased with increased BP-multiplications for all renal functions on the day of sampling. CRTA declined more pronounced in the worst-case scenario than in the pathogen-based scenario. When 4 × BP was targeted, only patients experiencing AKI III on the day of sampling in the pathogen-based scenario achieved a 100% CRTA. An evaluation of up to 6 × BP, as well as a breakdown of CRTA by non-steady or steady sample state can be found in the Additional file 1: Figs. S1, S2 and S3.

**Fig. 4:**
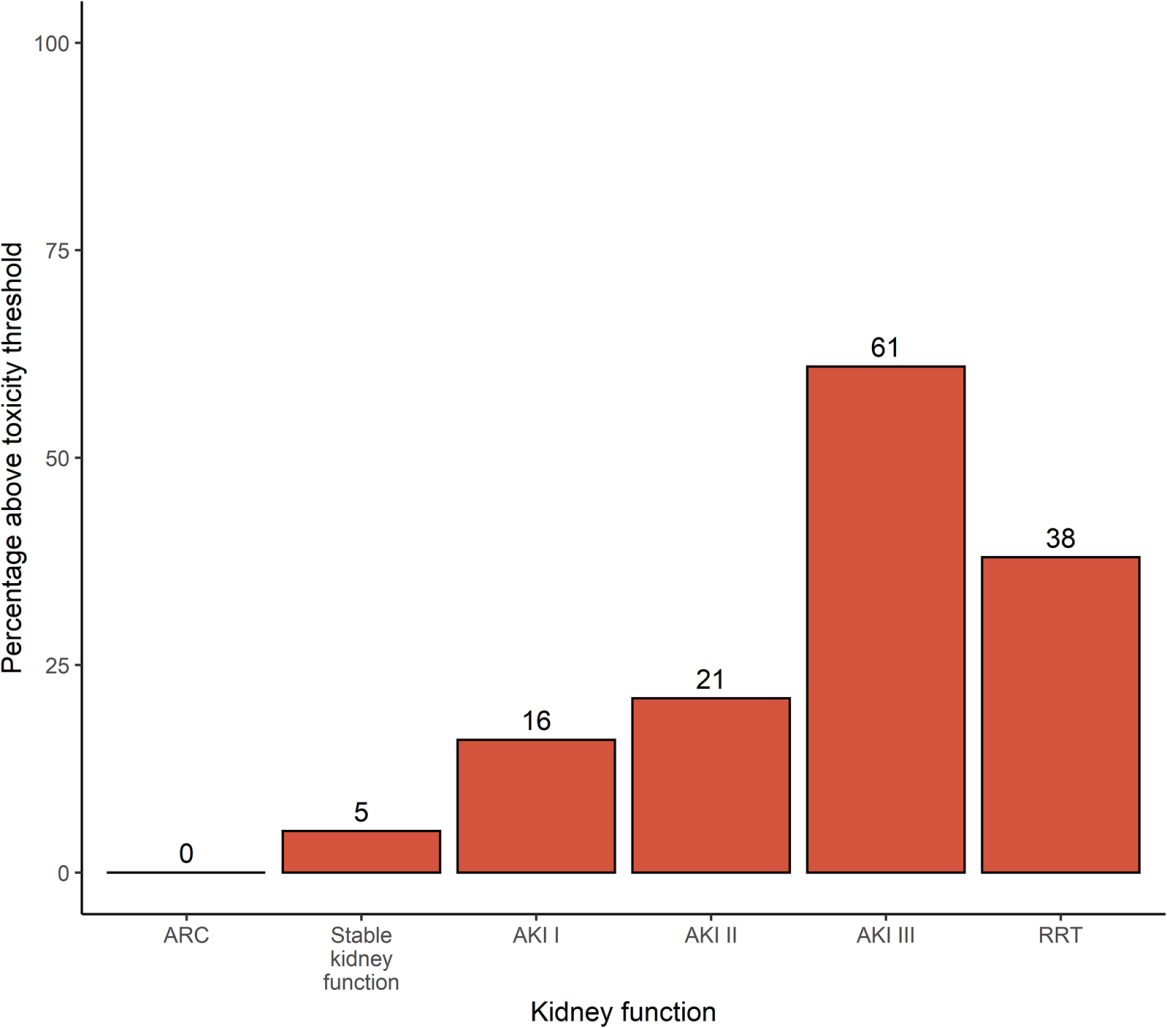
Percentage of samples above the toxicity threshold for TZP according to renal function on day of sampling

The number of samples (all measured samples, not only the ones for which a BP was available) exceeding the upper limit of the therapeutic range increased with declining renal function (see Fig. 4). With increasing stages of acute renal failure, the percentage of samples surpassing the toxicity thresholds increased.

**Fig. 5:**
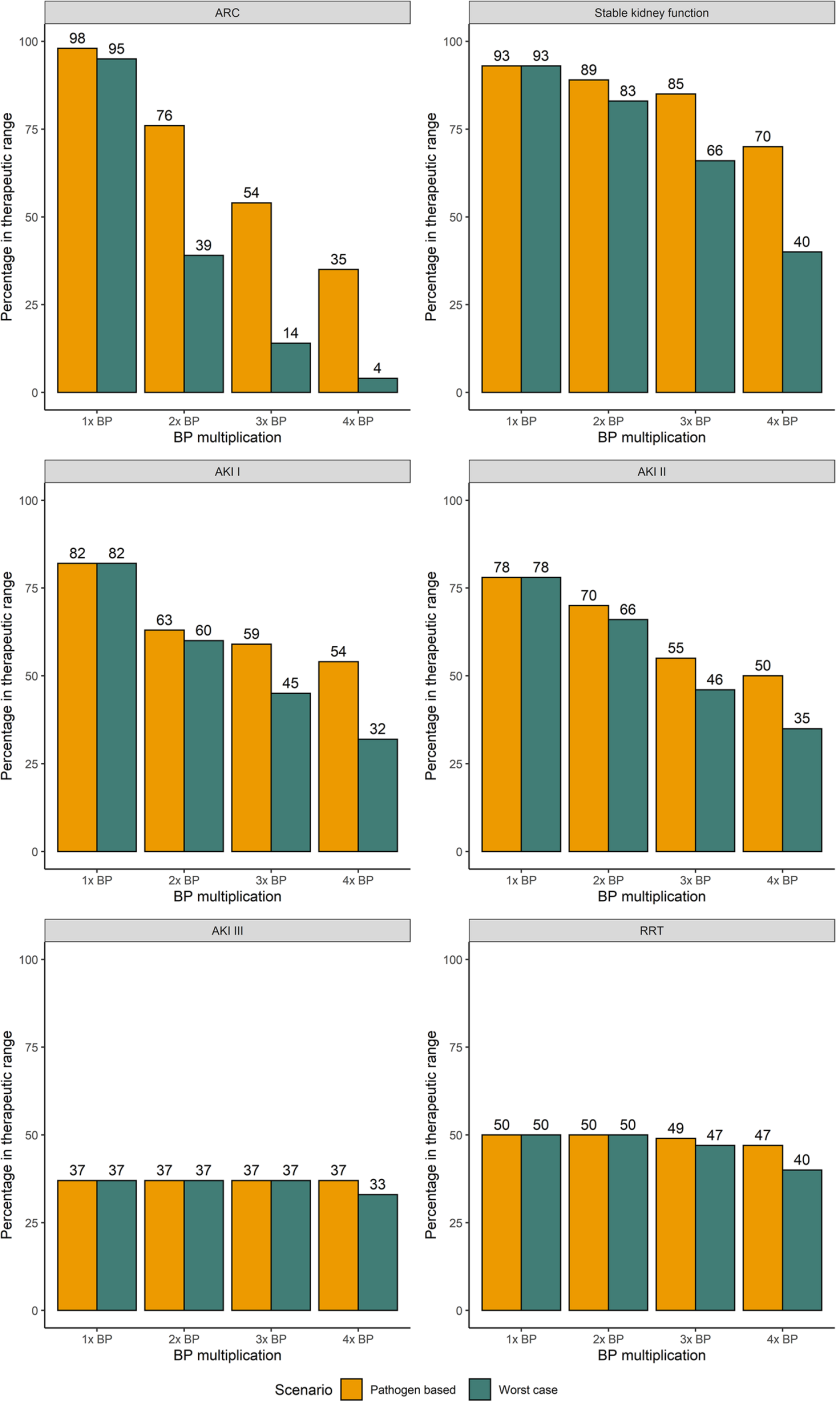
Percentage of samples within the therapeutic range for TZP according to the different BP multiplications and renal function

The comparison of samples falling within the therapeutic range between the pathogen-based setting and the worst-case scenario is shown in Fig. 5. In patients with AKI or RRT on the day of sampling, supratherapeutic levels contributed to a large part of samples falling outside the therapeutic range, whereas this was limited in patients with a normal kidney function or ARC. An evaluation of up to 6 × BP, as well as a breakdown of therapeutic range attainment by non-steady or steady sample state can be found in the Additional file 1: Figs. S4, S5 and S6.

#### Meropenem

**Fig. 6:**
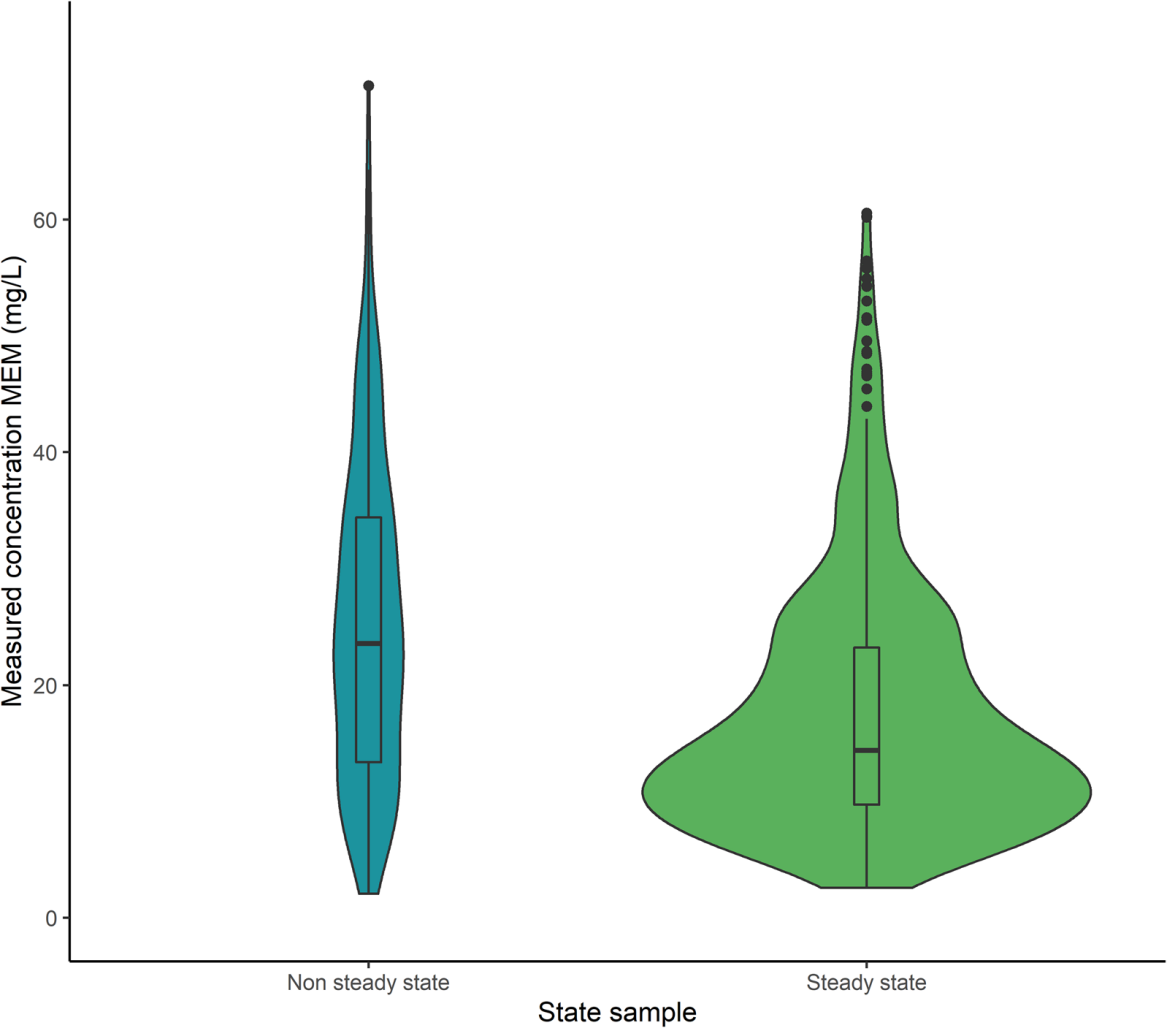
Violin plot of free MEM concentrations according to sample state (*p* = 0.23)

**Fig. 7:**
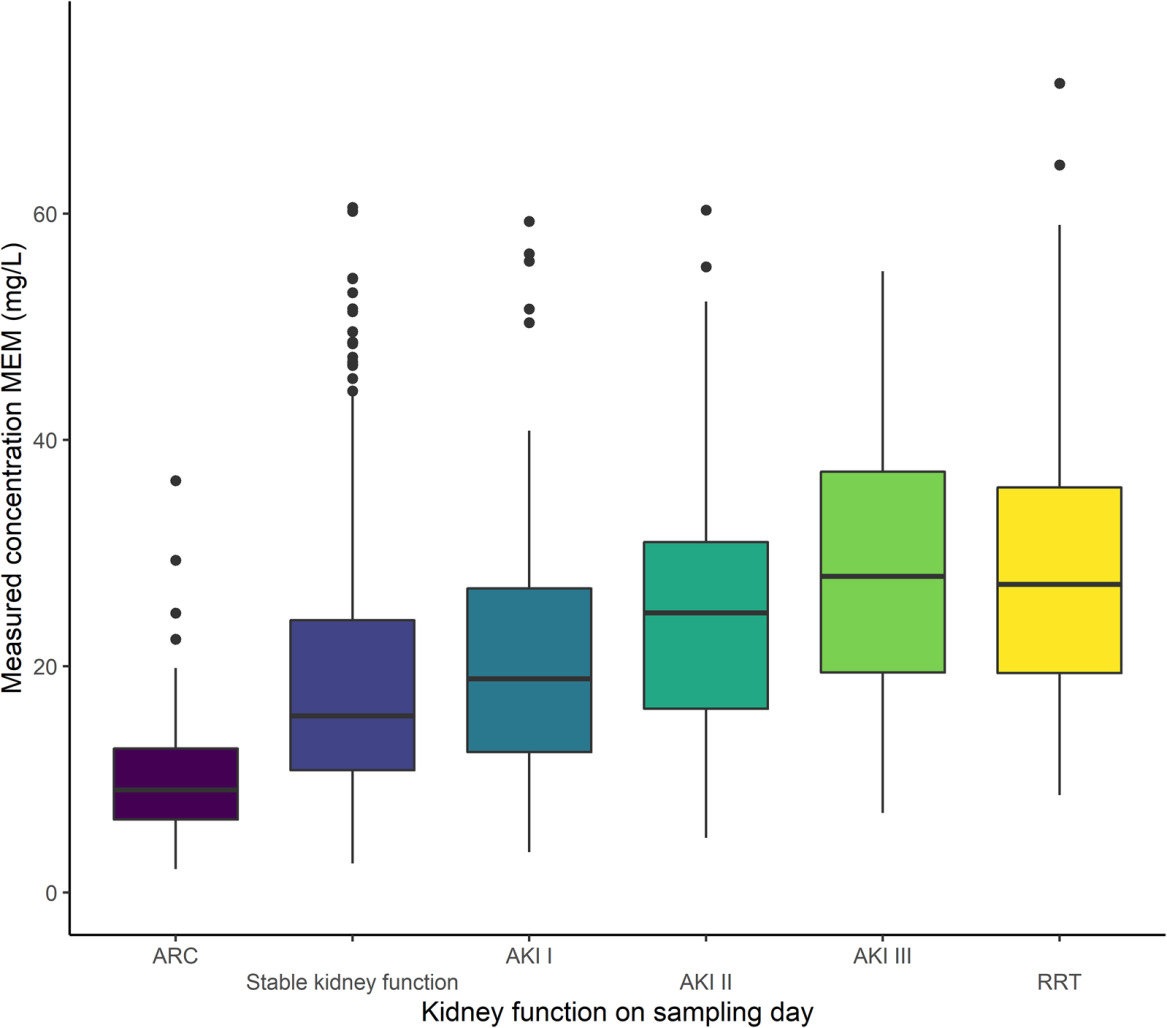
Boxplot of free MEM concentrations according to renal function on the sampling day

The median measured concentration for MEM was 23.7 mg/L [14.0–33.4] for non-steady state samples and 14.4 mg/L [9.75–22.89] for steady state samples (Fig. 6—*p* = 0.23). Distributions of measured concentrations according to the renal function are shown in Fig. 7. As for TZP, the lowest concentrations were measured when augmented renal clearance was present on the day of sampling, while increasingly higher concentrations were measured with increasing stages of acute kidney injury. Measured concentrations for RRT samples were again higher than when the renal function was stable.

**Fig. 8:**
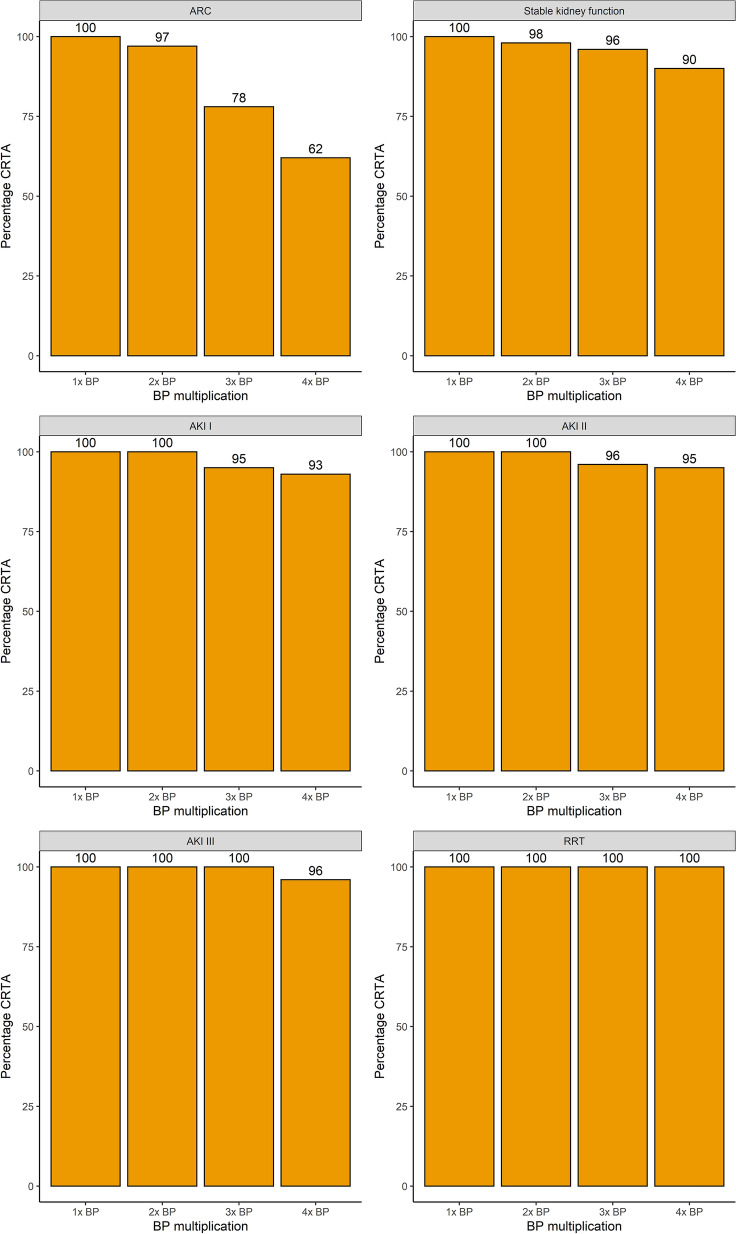
Evolution of percentage of MEM CRTA according to kidney function for different BP multiplications

The evolution of the percentage of clinically relevant target attainment for different BP multiplications with regards to the renal function on the day of sampling is shown in Fig. 8. An evaluation of up to 6 × BP, as well as a breakdown of CRTA by non-steady or steady sample state can be found in Additional file 1: Figs. S7, S8 and S9. For meropenem, a BP of 2 was the target for every sample for which a pathogen was identified, hence no distinction between a pathogen-based and worst-case scenario could be made. As for TZP, the number of samples reaching CRTA declined when a higher BP multiplication was targeted; this decline was more pronounced for patients with ARC or a stable renal function on the day of sampling than for patients with AKI or receiving RRT.

**Fig. 9:**
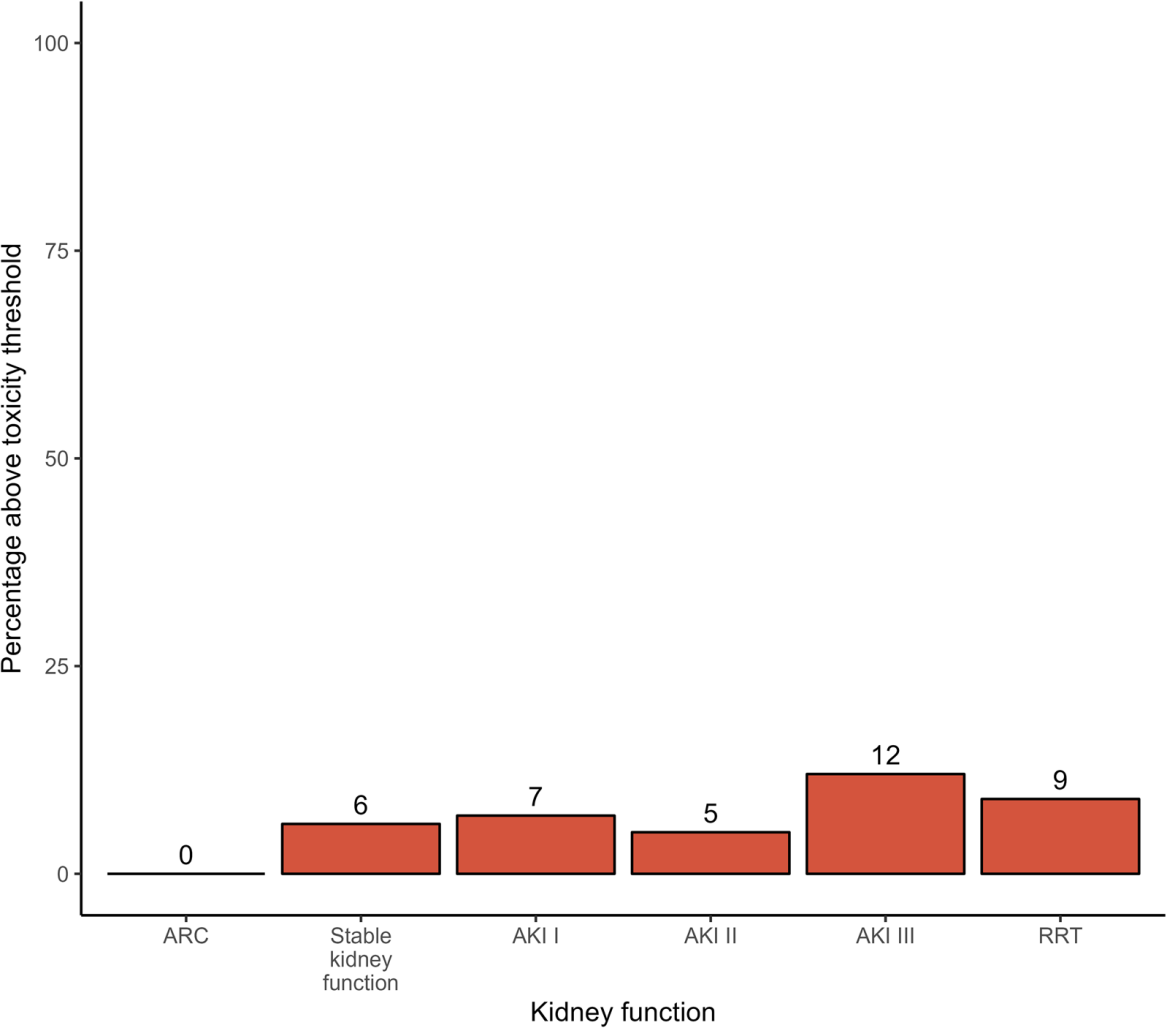
Percentage of MEM samples above the toxic threshold according to renal function

**Fig. 10:**
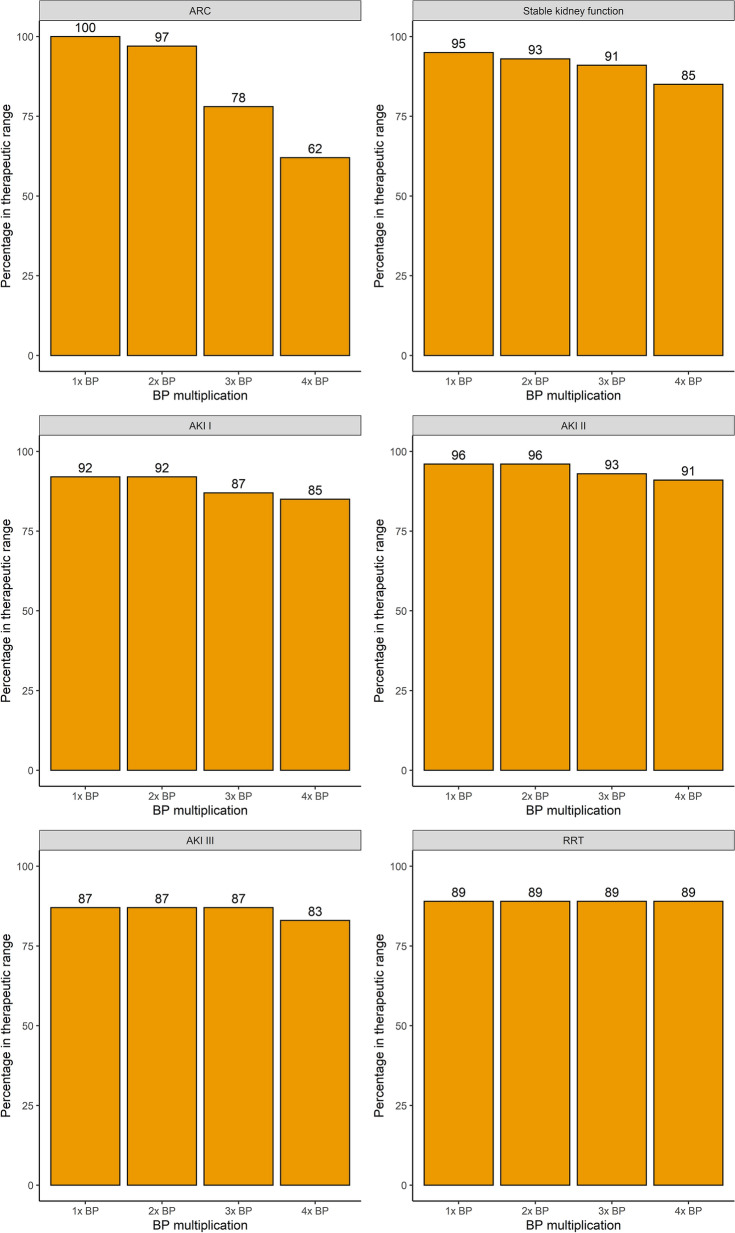
Percentage of MEM samples within the therapeutic range for different BP multiplications according to renal function

Most of the samples remained below the toxicity threshold (Fig. 9). When evaluating the percentage of samples within the therapeutic range, the effect of samples above the toxic threshold was less pronounced than with TZP (Fig. 10). An evaluation of up to 6 × BP, as well as a breakdown by non-steady and steady sample state is provided in Additional file 1: Figs. S10, S11 and S12.

## Discussion

In this extensive study cohort of surgical ICU patients, predominantly treated for a respiratory or abdominal infection with a limited proportion being in septic shock, we found highly variable concentrations for commonly used broad spectrum antimicrobials. Non-steady state concentrations were higher than steady state concentrations as 45% of these samples were taken either from patients who were admitted less than 12h to the ICU and in whom treatment was already initiated in the ward (where TZP/MEM is given as an extended infusion with a loading dose) or from patients who received a loading dose in the ICU. CRTA and TRA was more stable for MEM than for TZP across variations in renal function on the day of sampling. Pathogen-based clinically relevant target attainment and therapeutic range attainment was consistently higher than in the worst-case scenario. There was still an important percentage of pathogen-based non-attainment, which was higher for TZP samples than for MEM samples (e.g., for a targeted BP multiplication of 4 with a stable kidney function, target non-attainment was 24% for TZP while only 10% for MEM). The kidney function on the day of sampling, the BP of the pathogen and the targeted BP multiplication all contributed to this non-attainment. In the current study, the observed concentrations for TZP and MEM were comparable to previously reported concentrations. For TZP, other studies reported both higher and lower median concentrations, while for MEM higher median concentrations were frequently reported [19–23]. The crude percentages of over- and underdosing reported here are difficult to compare with other studies, as a multitude of therapeutic targets and ranges are used in the literature. The general trends (underdosing with increasing renal clearance and/or targeted BP multiplication, overdosing with decreasing renal function) are partially consistent with the findings of a similar study [24].

After the DALI-study revealed that underdosing of beta-lactam antimicrobials was common in critically ill patients, several dose optimization strategies, such as higher maintenance doses and the use of continuous infusions, were developed and implemented [25]. These strategies were also used during this study, resulting in almost all samples attaining the minimum—conservative—clinical target set forward by the joint ESICM/IATDMCT/ISAC position paper for beta-lactam antimicrobials given by continuous infusion: a measured steady state concentration above the BP [4]. However, despite the optimization strategies, a substantial proportion of TZP samples failed to reach the threshold of 4 × BP, which is considered the threshold to prevent microbiological failure and/or the development of resistance [26, 27]. Other approaches are needed to reach these targets. Strategies such as model informed precision dosing or the use of higher than conventional maintenance doses, for instance targeting a ‘maximum tolerable dose’, are, therefore, worth investigating [28]. For MEM, however, the proportion of samples that reached the threshold of 4 × BP was much higher than TZP when using a standard dosing regimen of 3g per 24h, except in patients with ARC. Although some ICUs use meropenem high dose as a standard of care, our findings and the advised standard regimens set out by EUCAST do not provide supportive evidence for this practice [29].

On the other side of the spectrum, overdosing and toxicity also remain as areas of uncertainty and concern. Renal toxicity and neurotoxicity are most feared during TZP and MEM treatment. Neurotoxicity is believed to be dose dependent, while renal toxicity due to tubulointerstitial nephritis is not. Investigating neurotoxicity in infected critically ill patients is difficult due to the plethora of possible confounders. Hence, to date, no definitive toxicity threshold has been identified. More research is, however, desirable, as the results of this study indicate a sizable effect of overdosing, and hence the toxicity threshold, on therapeutic range attainment. This effect is more pronounced for TZP than for MEM. The combined effect of over- and underdosing results in a substantial variability in pathogen-based therapeutic range attainment. This supports the joint recommendation of ESICM/IADTMDCT/ISAC to use therapeutic drug monitoring for beta-lactam treatment monitoring in critically ill patients to ascertain the attainment of PK/PD targets. From a clinical point of view, the findings of this study could give direction to clinicians when to consider therapeutic drug monitoring depending on the clinical status of the patient, the local resistance pattern, the kidney function of the patient and whether the physician wants to avoid under- or overdosing.

We introduced the concept of ‘pathogen-based scenario analysis’, based on the rationale that clinical relevant target attainment could be underestimated when a worst-case scenario is used. For MEM, there was no difference in CRTA between a pathogen-based and a worst-case scenario, as the BP of the identified pathogens and the worst-case scenario do not differ. For piperacillin, however, pathogen-based clinical relevant target attainment and therapeutic range attainment was consistently higher compared to a worst-case scenario, but still far from optimal, influenced by the renal function of the patient or the targeted BP multiplication. To our knowledge, there is only one comparable study by Weinelt et al. that evaluated a drug concentration monitoring program for MEM and TZP [24]. In this smaller study (patients: 108 MEM–96 TZP, samples: 375 MEM–230 TZP) a MIC value could be determined for 53 patients receiving MEM and 33 patients receiving TZP. The determined MIC was lower than 2 mg/L in 79.2% of MEM cases and lower than 16 mg/L in 93.9% of TZP cases, resulting in a higher clinical relevant target attainment for pathogen-based samples than for samples for which a worst-case scenario was used for both MEM and TZP. These consistent findings could potentially have implications for the interpretation of recently conducted trials that evaluate the impact of therapeutic drug monitoring on clinical outcomes [5, 30]. In the TARGET-trial, no causative pathogen was identified for 34.4% of patients, while in the DOLPHIN-trial a worst-case scenario was assumed for all patients treated with piperacillin-tazobactam. Both studies did not show a statistical benefit of the use of TDM on clinical primary endpoints. Both our study and the study by Weinelt et al. indicate that the used methodology could underestimate the clinically relevant target attainment, which could in turn influence the results of these trials. While from a clinical point of view, there is no denying that a worst-case scenario needs to be assumed when starting empiric antimicrobial therapy, a pathogen-based approach might prove to be more sensible when evaluating target attainment and linked clinical outcomes. Ideally, future research uses a causative pathogen-based approach, albeit that MIC determination of individual pathogens has important limitations as well [31]. The BP method used in this work and by others might be considered as an alternative [5].

Strengths of this study include the scale and granularity of the collected data, the use of optimized dosing regimens by means of continuous infusion and the extent and variety of included patients. Some limitations must be acknowledged. As our microbiology laboratory does not routinely determine MIC measurements for identified pathogens, BPs were used. Secondly, both steady state and non-steady state samples were used for target attainment calculation. For MEM, the median concentration was not statistically different between steady and non-steady state samples. A statistical difference was found for TZP, however, the impact on the results was only modest as illustrated by Additional file 1: Figs. S1–S6. It was, therefore, felt justified to include all samples for the analysis. Third, a 30% protein binding of TZP was assumed. This percentage is based on information provided by the drug manufacturer. However, conflicting evidence emerged in recent years, with median protein binding percentages ranging from 9 to 52% [32, 33]. As the free fraction is the biologically active fraction of a drug and the assumed protein binding impacts the dosing recommendation, more research into the real-world protein binding of piperacillin in ICU patients seems warranted to further optimize antimicrobial care. Fourth, as the CRTA and TRA analysis are dependent on the breakpoint of the identified pathogen, the results of these analyses are only valid for pathogens susceptible to the investigated antimicrobials. Finally, the study design did not permit us to formally evaluate the effect of antimicrobial concentrations on endpoints in a rigorous manner. Interestingly enough, a similar U-shaped mortality curve was found as in the TARGET-trial by means of a crude analysis. However, several caveats apply. This is explored more in depth in the Additional file 1.

## Conclusion

Despite pathogen-based data indicating that clinical relevant target attainment and therapeutic range attainment is higher than in the often-used theoretical worst-case scenario, a substantial proportion of samples did not attain commonly used PK/PD targets when using optimised continuous infusion dosing regimens, supporting the use of therapeutic drug monitoring during TZP and MEM treatment. For clinical research, a ‘pathogen-based analysis’ approach might prove to be more sensible than a worst-case scenario approach when evaluating target attainment and linked clinical outcomes.

## Supplementary Information

**Additional file1: Table S1**. Distribution of infection foci as a percentage of total infections treated. **Table S2**. Top 10 most frequently identified pathogens. **Table S3**. Clinical characteristics of the collected samples [counts and]. **Table S4**. Evaluation of dosing appropriateness [count and]. **Table S5**. Evaluation of dosing appropriateness according to AKI stage on sampling day [counts and]. **Figure S1**. Percentage of CRTA for all TZP samples according to renal function and BP multiplication targeted. **Figure S2**. Percentage of CRTA for TZP steady state samples according to renal function and BP multiplication targeted. **Figure S3**. Percentage of CRTA for TZP non-steady state samples according to renal function and BP multiplication targeted. **Figure S4**. Percentage of all TZP samples within the therapeutic range for different BP multiplications according to the BP and renal function. **Figure S5**. Percentage of steady state TZP samples within the therapeutic range for different BP multiplications according to the BP and renal function. **Figure S6**. Percentage of non-steady state TZP samples within the therapeutic range for different BP multiplications according to the BP and renal function. **Figure S7**. Percentage of CRTA for all MEM samples according to renal function and BP multiplication. **Figure S8**. Percentage of CRTA for MEM steady state samples according to renal function and BP multiplication. **Figure S9**. Percentage of CRTA for MEM non-steady state samples according to renal function and BP multiplication. **Figure S10**. Percentage of all MEM samples within the therapeutic range for different BP multiplications according to the BP and renal function. **Figure S11**. Percentage of steady state MEM samples within the therapeutic range for different BP multiplications according to the BP and renal function. **Figure S12**. Percentage of non-steady state MEM samples within the therapeutic range for different BP multiplications according to the BP and renal function.

## Data Availability

The datasets generated and/or analyzed during the current study are available from the corresponding author upon reasonable request.

## Abbreviations

AKI: Acute kidney injury
AKI I: Acute kidney injury stage I according to KDIGO
AKI II: Acute kidney injury stage II according to KDIGO
AKI III: Acute kidney injury stage III according to KDIGO
ARC: Augmented renal clearance
BP: Break point
CKD: Chronic kidney dysfunction
CKD-EPI: Chronic Kidney Disease–Epidemiology Collaboration
COSARA: Computer-based surveillance and alerting of nosocomial infections, antimicrobial resistance and antibiotic consumption in the ICU
CRTA: Clinically relevant target attainment
ECOFF: Epidemiological cut-off value
ESCMID: European Society of Clinical Microbiology and Infectious Diseases
ESICM: European Society of Intensive Care Medicine
EUCAST: European Committee on Antimicrobial Susceptibility Testing
IATDMCT: International Association for Therapeutic Drug Monitoring and Clinical Toxicology
ICU: Intensive Care Unit
ISAC: International society of antimicrobial chemotherapy
KDIGO: Kidney disease, improving global outcomes
MEM: Meropenem
PK/PD: Pharmacokinetic/Pharmacodynamic
RRT: Renal replacement therapy
SFM: Société Française de Microbiologie
TDM: Therapeutic drug monitoring
TRA: Therapeutic range attainment
TZP: Piperacillin-tazobactam
UPLC-MS/MS: Ultra-performance liquid chromatographic with tandem mass spectrometry
