# Supplementary material for: Pathogen-based target attainment of optimized continuous infusion dosing regimens of piperacillin-tazobactam and meropenem in surgical ICU patients: a prospective single center observational study
Source: Ann Intensive Care. 2023 Apr 29;13:35. doi: 10.1186/s13613-023-01129-6 (PMC10148758; doi:10.1186/s13613-023-01129-6)
Supplement: Supplementary file 1 — Additional file1: Table S1. Distribution of infection foci as a percentage of total infections treated. Table S2. Top 10 most frequently identified pathogens. Table S3. Clinical characteristics of the collected samples [counts and]. Table S4. Evaluation of dosing appropriateness [count and]. Table S5. Evaluation of dosing appropriateness according to AKI stage on sampling day [counts and]. Figure S1. Percentage of CRTA for all TZP samples according to renal function and BP multiplication targeted. Figure S2. Percentage of CRTA for TZP steady state samples according to renal function and BP multiplication targeted. Figure S3. Percentage of CRTA for TZP non-steady state samples according to renal function and BP multiplication targeted. Figure S4. Percentage of all TZP samples within the therapeutic range for different BP multiplications according to the BP and renal function. Figure S5. Percentage of steady state TZP samples within the therapeutic range for different BP multiplications according to the BP and renal function. Figure S6. Percentage of non-steady state TZP samples within the therapeutic range for different BP multiplications according to the BP and renal function. Figure S7. Percentage of CRTA for all MEM samples according to renal function and BP multiplication. Figure S8. Percentage of CRTA for MEM steady state samples according to renal function and BP multiplication. Figure S9. Percentage of CRTA for MEM non-steady state samples according to renal function and BP multiplication. Figure S10. Percentage of all MEM samples within the therapeutic range for different BP multiplications according to the BP and renal function. Figure S11. Percentage of steady state MEM samples within the therapeutic range for different BP multiplications according to the BP and renal function. Figure S12. Percentage of non-steady state MEM samples within the therapeutic range for different BP multiplications according to the BP and renal function. [file 13613_2023_1129_MOESM1_ESM.docx]

**Real-world target attainment of optimized continuous infusion dosing regimens of piperacillin – tazobactam and meropenem in surgical ICU patients - a prospective observational study**

**-**

Additional file 1

T. De Corte, J. Verhaeghe, S. Dhaese, S. Van Vooren, J. Boelens, A. G. Verstraete, V. Stove, F. Ongenae, L. De Bus, P. Depuydt, S. Van Hoecke, J.J. De Waele

**TABLES**

| OS Table 1 - Distribution of infection foci as a percentage of total infections treated | |
| --- | --- |
| **Infection focus** | **Count *(percentage)*** |
| Respiratory infection | 345 (34.4) |
| Abdominal infection | 343 (34.2) |
| Prophylaxis | 114 *(11.4)* |
| Skin and soft tissue infection | 62 (6.18) |
| Unkown infection site | 50 (4.90) |
| Urinary tract infection | 28 (2.79) |
| Endocarditis or intravascular infection | 22 *(2.19)* |
| Central line associated blood stream infection | 17 (1.69) |
| Other infection | 11 (1.1) |
| Neurological infection | 7 (0.7) |
| Neutropenic sepsis | 4 (0.4) |

| OS Table 2 - Top 10 most frequently identified pathogens | | | |
| --- | --- | --- | --- |
| **Pathogen identified per unique infection** | **Count *(percentage)*** | **BP TZP** | **BP MEM** |
| 1. Escherichia coli | 87 *(23.8)* | 8 | 2 |
| 2. Enterobacter cloacae | 45 (12.3%) | 8 | 2 |
| 3. Pseudomonas aeruginosa | 43 (11.8%) | 16 | 2 |
| 4. Klebsiella pneumoniae | 25 (6.8%) | 8 | 2 |
| 5. Staphylococcus aureus | 23 (6.3%) | NA | NA |
| 6. Klebsiella oxytoca | 14 (3.8%) | 8 | 2 |
| 7. Klebsiella aerogenes | 11 (3.0%) | 8 | 2 |
| 8. Serratia marescens | 11 (3.0%) | 8 | 2 |
| 9. Enterococcus faecalis | 10 (2.73%) | NA | NA |
| 10. Proteus mirabilis | 7 (1.91%) | 8 | 2 |
| *BP = Break point. NA = Not available* | | | |

| OS Table 3 - Clinical characteristics of the collected samples (counts and *(percentages)*) | | | | | | |
| --- | --- | --- | --- | --- | --- | --- |
| **Characteristic** | **Piperacillin-tazobactam** | | | **Meropenem** | | |
|  | **Steady state** | **Non steady state** | **All samples** | **Steady state** | **Non steady state** | **All samples** |
|  | **(N = 1,462)** | **(N = 430)** | **(N = 1,892)** | **(N = 709)** | **(N = 209)** | **(N = 918)** |
| **Renal function** |  |  |  |  |  |  |
| Augmented Renal Clearance | 331 (*22.6)* | 49 (*11.4)* | 380 *(20.1)* | 156 *(22.0)* | 22 *(10.5)* | 178 *(19.4)* |
| Stable renal function | 660 (*45.1)* | 148 *(34.4)* | 808 *(42.7)* | 366 *(51.6)* | 47 *(22.5)* | 413 *(45.)* |
| AKI I | 182 *(12.4)* | 52 *(12.1)* | 234 *(12.4)* | 76 *(10.7)* | 21 *(10.0)* | 97 *(10.6)* |
| AKI II | 149 *(10.2)* | 39 *(9.1)* | 188 *(9.9)* | 48 *(6.8)* | 22 *(10.5 )* | 70 *(7.6)* |
| AKI III | 47 (3.2) | 16 *(3.7)* | 63 *(3.3)* | 16 *(2.3)* | 8 *(3.8)* | 24 *(2.6)* |
| RRT | 93 *(6.4)* | 126 *(29.3)* | 219 *(11.6)* | 47 *(6.6)* | 89 *(42.6)* | 136 *(14.8)* |
| **Infection focus** |  |  |  |  |  |  |
| Abdominal infection | 519 *(35.5)* | 158 *(36.7)* | 677 *(35.8)* | 325 *(45.8)* | 106 *(50.7)* | 431 *(46.9)* |
| Respiratory infection | 475 *(32.5)* | 109 *(25.3)* | 584 *(30.9)* | 174 *(24.5)* | 24 *(11.5)* | 198 *(21.6)* |
| Prophylaxis | 105 *(7.2)* | 45 *(10.5)* | 150 *(7.9)* | 14 *(2.0)* | 7 *(3.3)* | 21 *(2.3)* |
| Multiple concomitant infection foci | 109 *(7.5)* | 22 *(5.1)* | 131 *(6.9)* | 89 *(12.6)* | 31 *(14.8)* | 120 *(13.1)* |
| Skin and soft tissue infection | 74 *(5.1)* | 16 *(3.7)* | 90 *(4.8)* | 17 *(2.4)* | 9 *(4.3)* | 26 *(2.8)* |
| Endocarditis or intravascular infection | 31 *(2.1)* | 12 *(2.8)* | 43 *(2.3)* | 14 *(2.0)* | 10 *(4.8)* | 24 *(2.6)* |
| Central line associated blood stream infection | 14 *(1.0)* | 4 *(0.9)* | 18 *(1.0)* | 5 *(0.7)* | 3 *(1.4)* | 8 *(0.9)* |
| Neurological infection | 0 *(0.0)* | 2 *(0.5)* | 2 *(0.1)* | 11 *(1.6)* | 3 *(1.4)* | 14 *(1.5)* |
| Neutropenic sepsis | 7 *(0.5)* | 0 *(0.0)* | 7 *(0.4)* | 1 *(0.1)* | 0 *(0.0)* | 1 *(0.1)* |
| Other | 25 *(1.7)* | 10 *(2.3)* | 35 *(1.8)* | 3 *(0.4)* | 1 *(0.5)* | 4 *(0.4)* |
| Urinary tract infection | 12 *(0.8)* | 20 *(4.7)* | 32 *(1.7)* | 10 *(1.4)* | 5 *(2.4)* | 15 *(1.6)* |
| Unknown location | 91 *(6.2)* | 32 *(7.4)* | 123 *(6.5)* | 46 *(6.5)* | 10 *(4.8)* | 56 *(6.1)* |
| **Highest BP of the identified pathogen** |  |  |  |  |  |  |
| No pathogen identified or BP unknown | 606 *(41.4)* | 211 *(49.0)* | 817 *(43.2)* | 232 *(32.7)* | 65 *(31.1)* | 297 *(32.3)* |
| 0.25 mg/L | 8 *(0.6)* | 2 *(0.5)* | 10 *(0.5)* | - | - | - |
| 2 mg/L | - | - | - | 477 *(67.3)* | 144 *(68.9)* | 621 *(67.7)* |
| 8 mg/L | 637 *(43.6)* | 171 *(39.8)* | 808 *(42.7)* | - | - | - |
| 16 mg/L | 211 *(14.4)* | 46 *(10.7)* | 257 *(13.6)* | - | - | - |

| OS Table 4: Evaluation of dosing appropriateness (count and *(% of evaluable samples)*) | |  |
| --- | --- | --- |
|  | **Piperacillin - Tazobactam** | **Meropenem** |
|  | **(Number of evaluable samples = 1,671)** | **(Number of evaluable samples = 834)** |
| **Dosing in accordance with guideline** | 1,396 *(83.54%)* | 754 *(90.41%)* |
| **Dosing higher than advised by guideline** | 155 *(9.28%)* | 58 *(6.95%)* |
| **Dosing lower than advised by guideline** | 120 *(7.18%)* | 22 *(2.64%)* |

| OS Table 5: Evaluation of dosing appropriateness according to AKI stage on sampling day (counts and *(% of evaluable samples)* ) | | | |
| --- | --- | --- | --- |
| **Piperacillin-Tazobactam** | | | |
|  | **Dosing in accordance with guideline** | **Dosing higher than advised by guideline** | **Dosing lower than advised by guideline** |
| **ARC (N = 351)** | 315 *(89.74%)* | 17 *(4.84%)* | 19 *(5.41%)* |
| **Stable Kidney function (N = 700** | 619 *(88.43%)* | 30 *(4.29%)* | 51 *(7.29%)* |
| **AKI I (N = 188)** | 158 *(84.04%)* | 21 *(11.17%)* | *9 (4.79%)* |
| **AKI II (N = 160)** | 122 *(76.25%)* | 23 *(14.37%)* | 15 *(9.38%)* |
| **AKI III (N = 58)** | 30 *(51.72%)* | 19 *(32.76%)* | 9 *(15.52%)* |
| **RRT (N = 214)** | 152 *(71.03%)* | 45 *(21.03%)* | 17 *(7.94%)* |
| **Meropenem** | | | |
|  | **Dosing in accordance with guideline** | **Dosing higher than advised by guideline** | **Dosing lower than advised by guideline** |
| **ARC (N = 174)** | **172 (98.85%)** | **0 (0.0%)** | **2 (1.15%)** |
| **Stable Kidney function (N = 386)** | **348 (90.16%)** | **29 (7.51%)** | **9 (2.33%)** |
| **AKI I (N = 80)** | **62 (77.50%)** | **13 (16.25%)** | **5 (6.25%)** |
| **AKI II (N = 59)** | **50 (84.75%)** | **8 (13.56%)** | **1 (1.69%)** |
| **AKI III (N = 22)** | **10 (45.45%)** | **7 (31.82%)** | **4 (22.73%)** |
| **RRT (N = 113)** | **112 (99.12%)** | **1 (0.88%)** | **0 (0.0%)** |

**FIGURES**


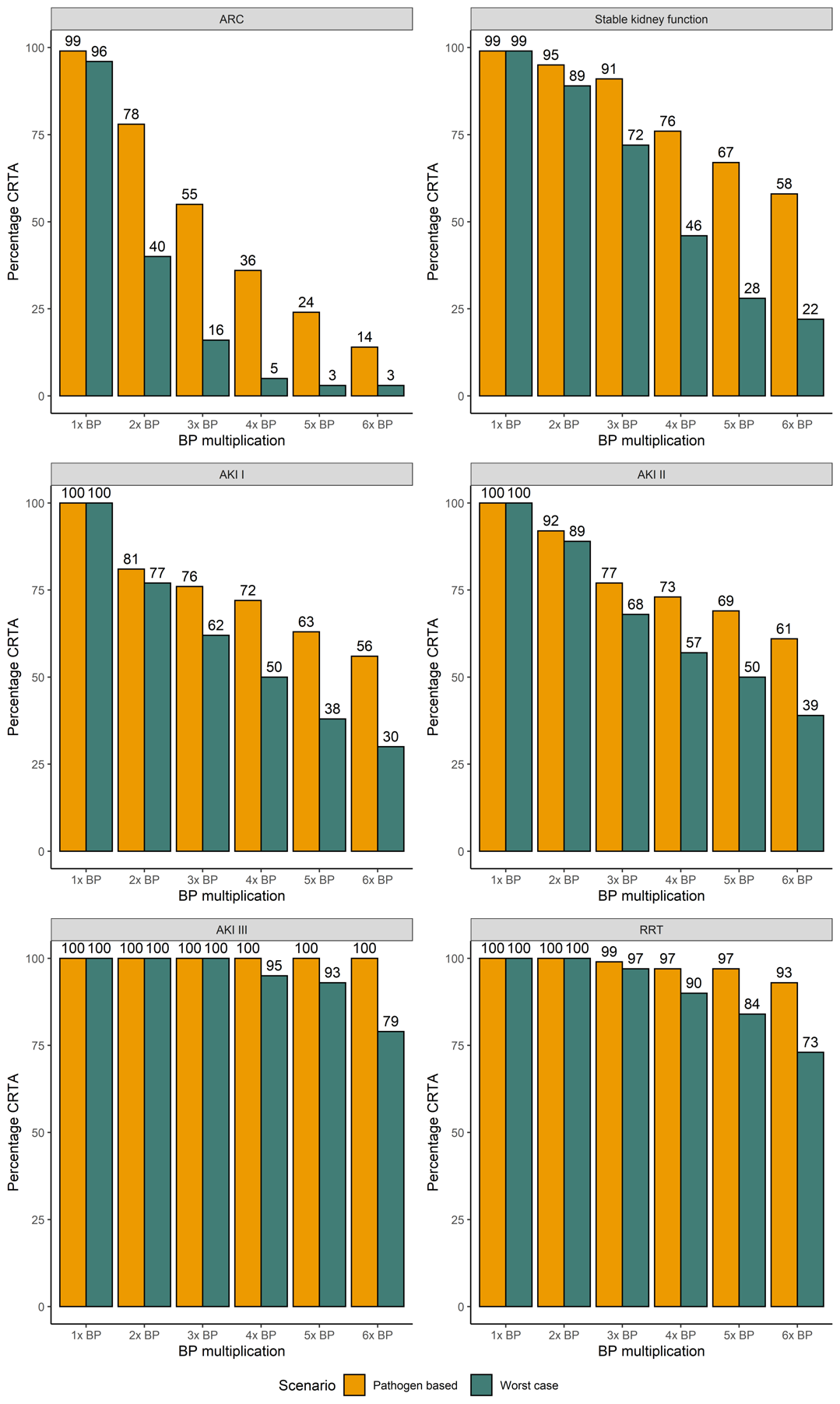

OS Figure 1 – Percentage of CRTA for all TZP samples according to renal function and BP multiplication targeted.


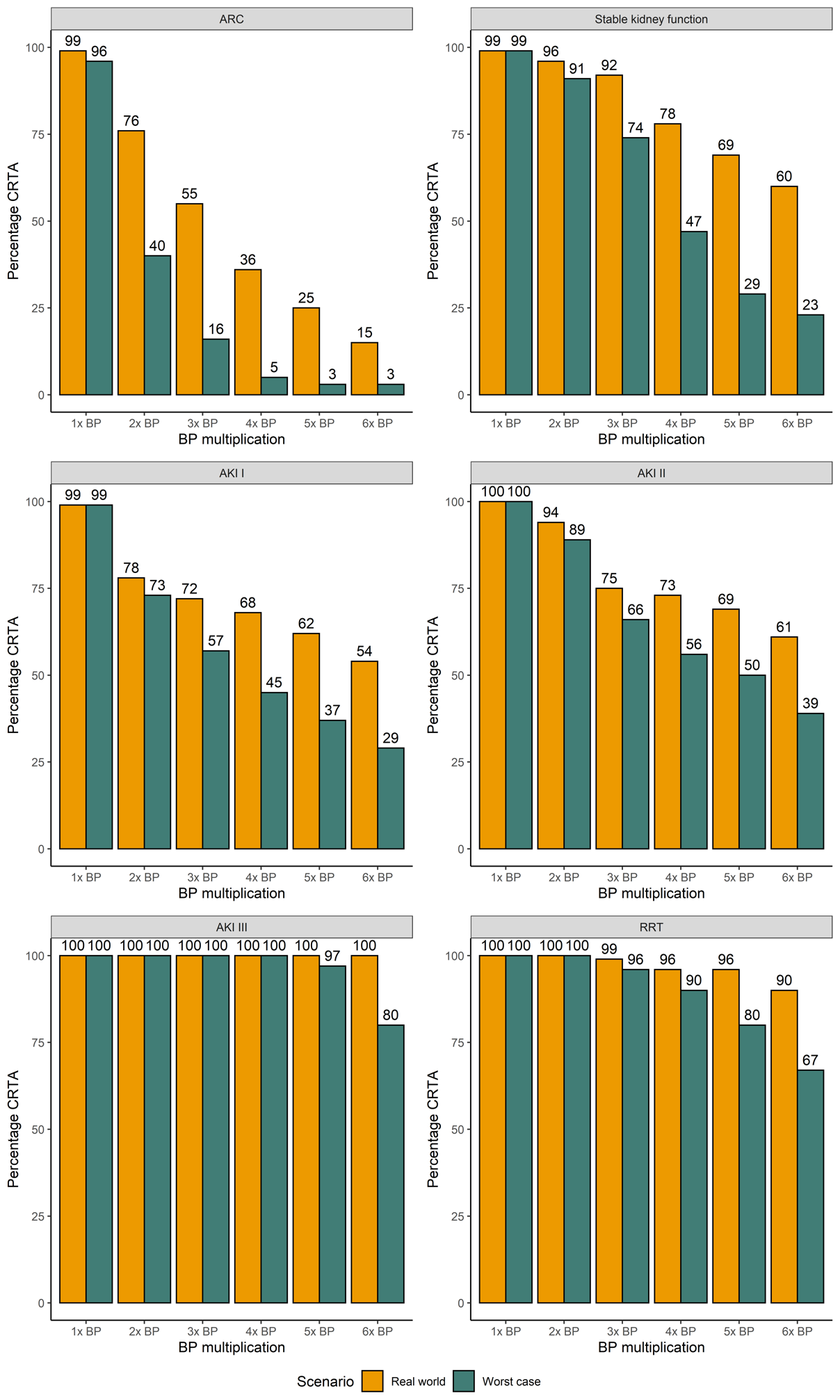


OS Figure 2 – Percentage of CRTA for TZP steady state samples according to renal function and BP multiplication targeted.


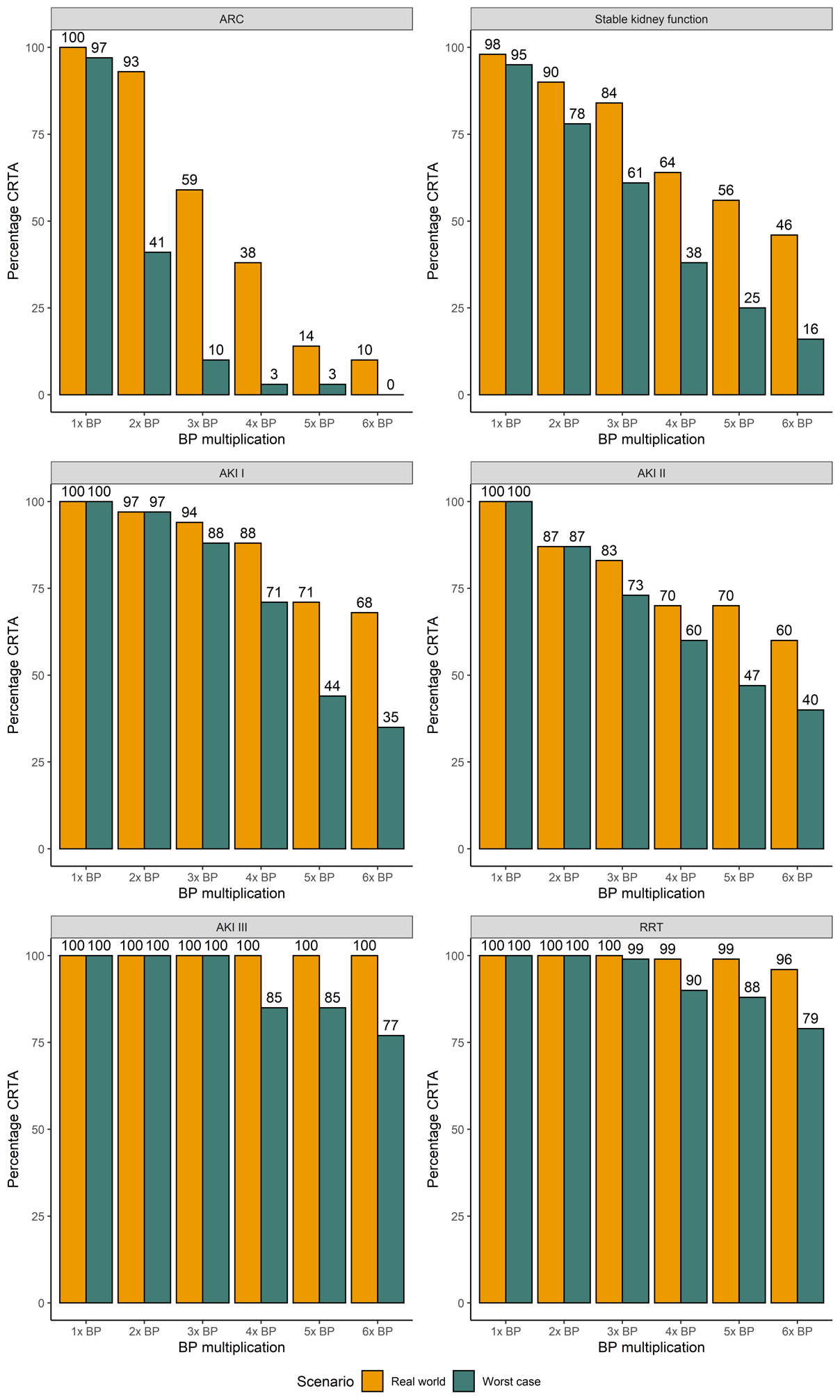


OS Figure 3 – Percentage of CRTA for TZP non-steady state samples according to renal function and BP multiplication targeted.


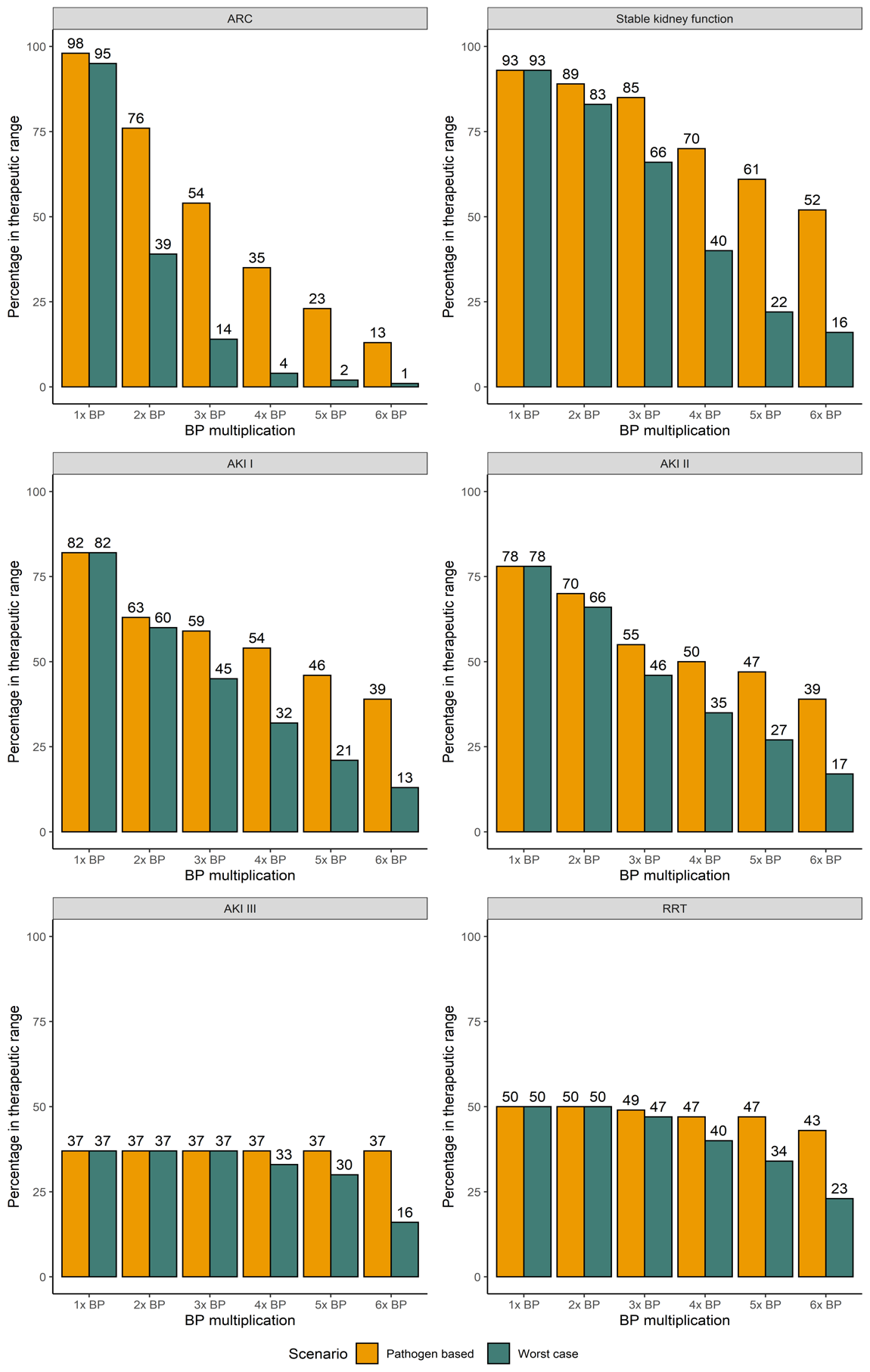


OS Figure 4: Percentage of all TZP samples within the therapeutic range for different BP multiplications according to the BP and renal function


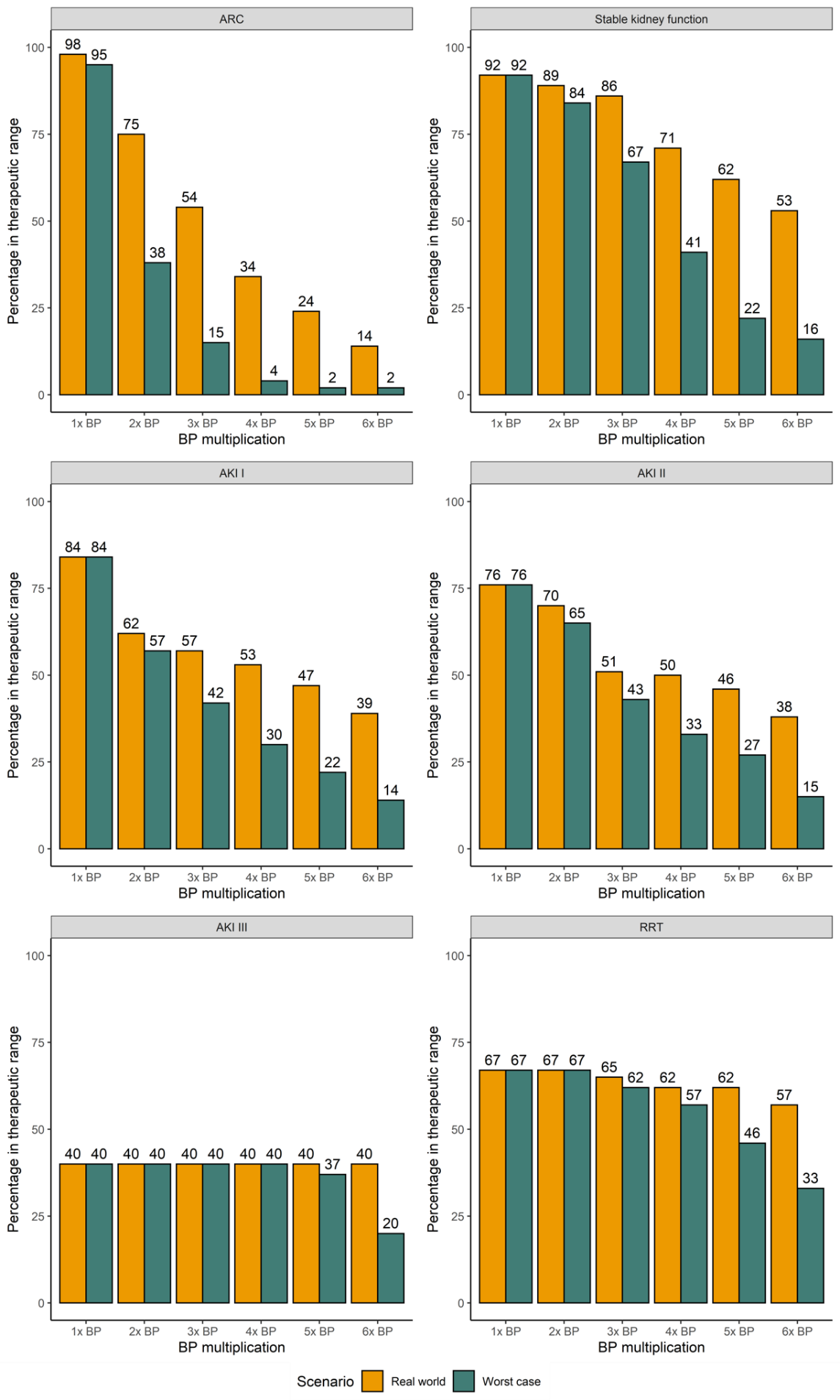


OS Figure 5: Percentage of steady state TZP samples within the therapeutic range for different BP multiplications according to the BP and renal function


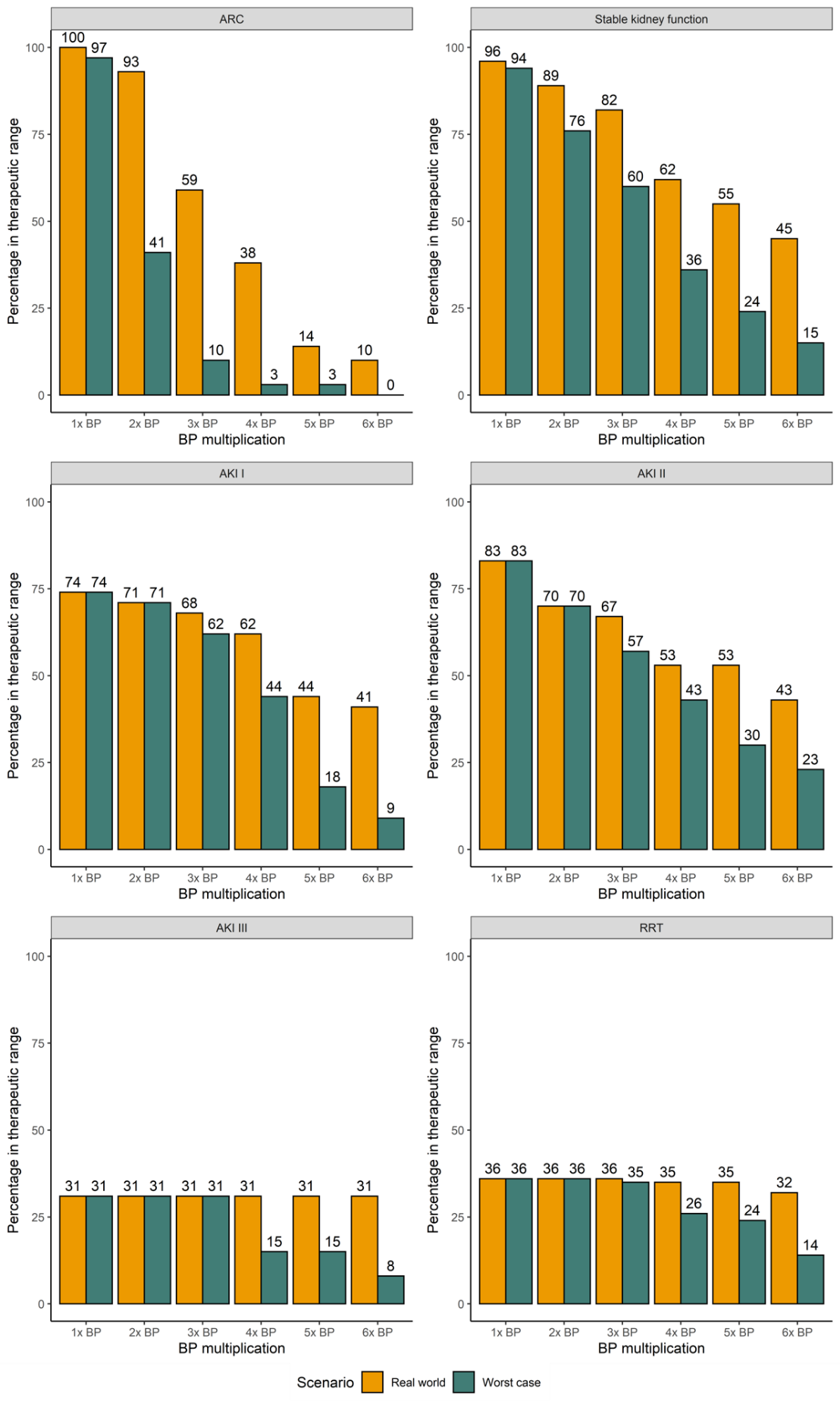


OS Figure 6: Percentage of non-steady state TZP samples within the therapeutic range for different BP multiplications according to the BP and renal function


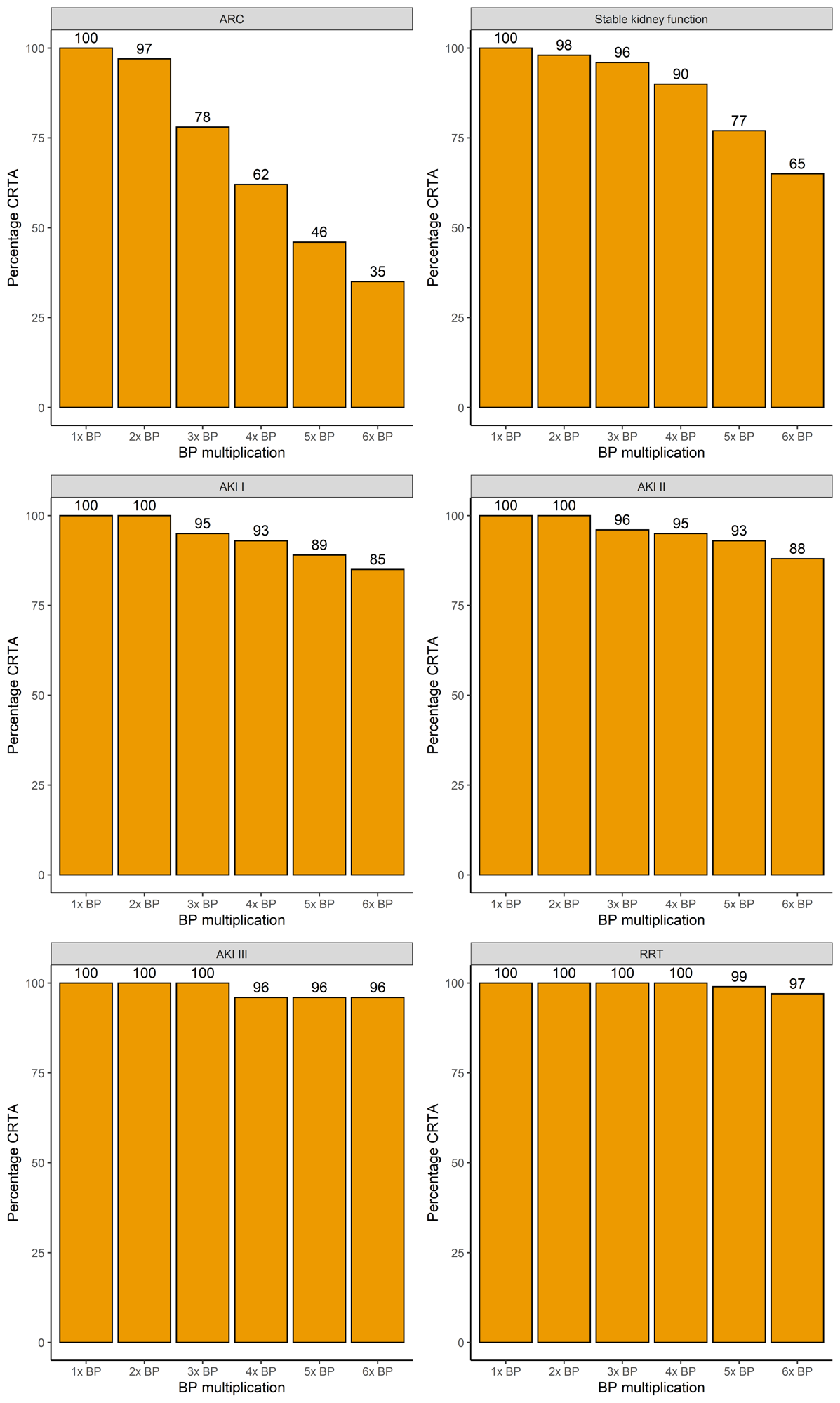


OS Figure 7 – Percentage of CRTA for all MEM samples according to renal function and BP multiplication


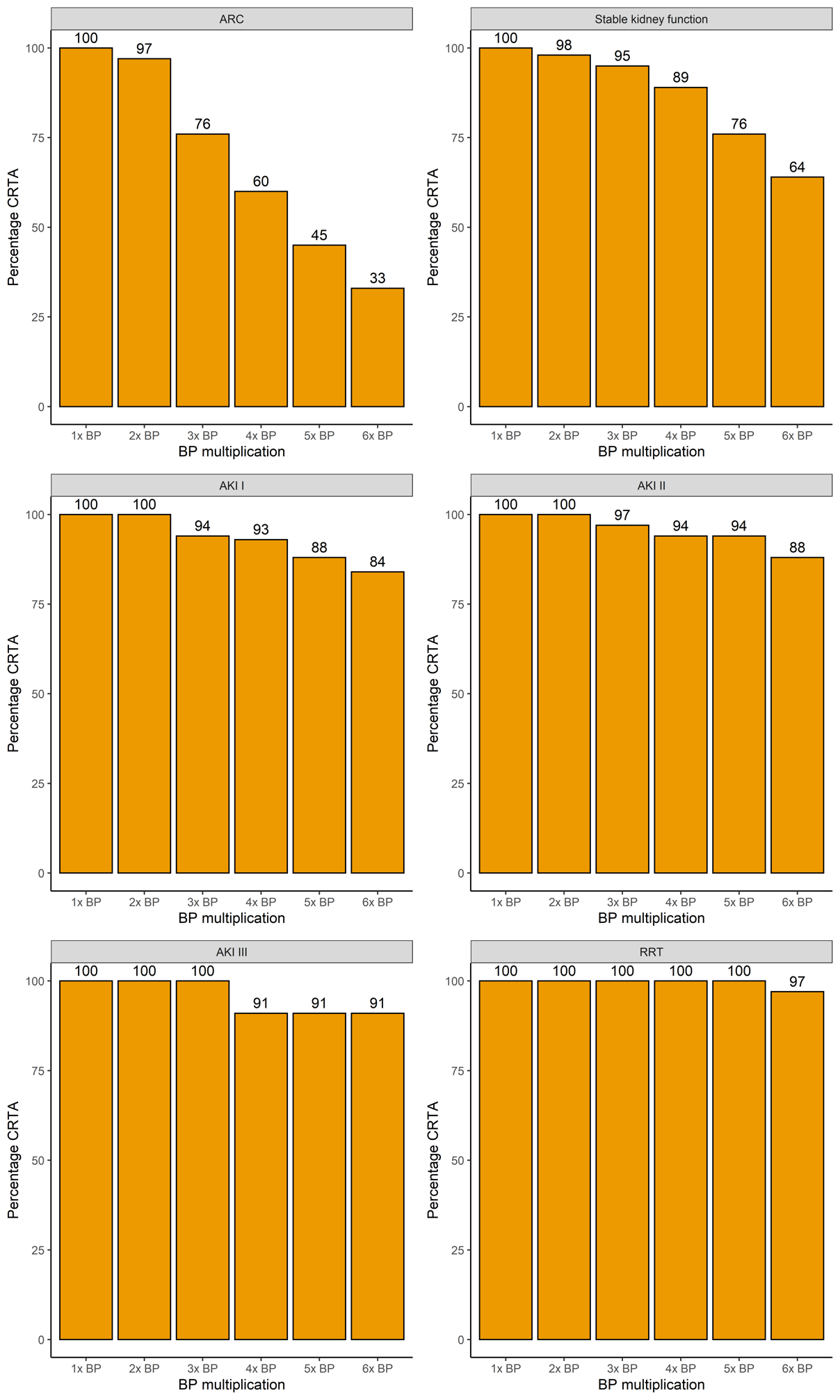


OS Figure 8 – Percentage of CRTA for MEM steady state samples according to renal function and BP multiplication.


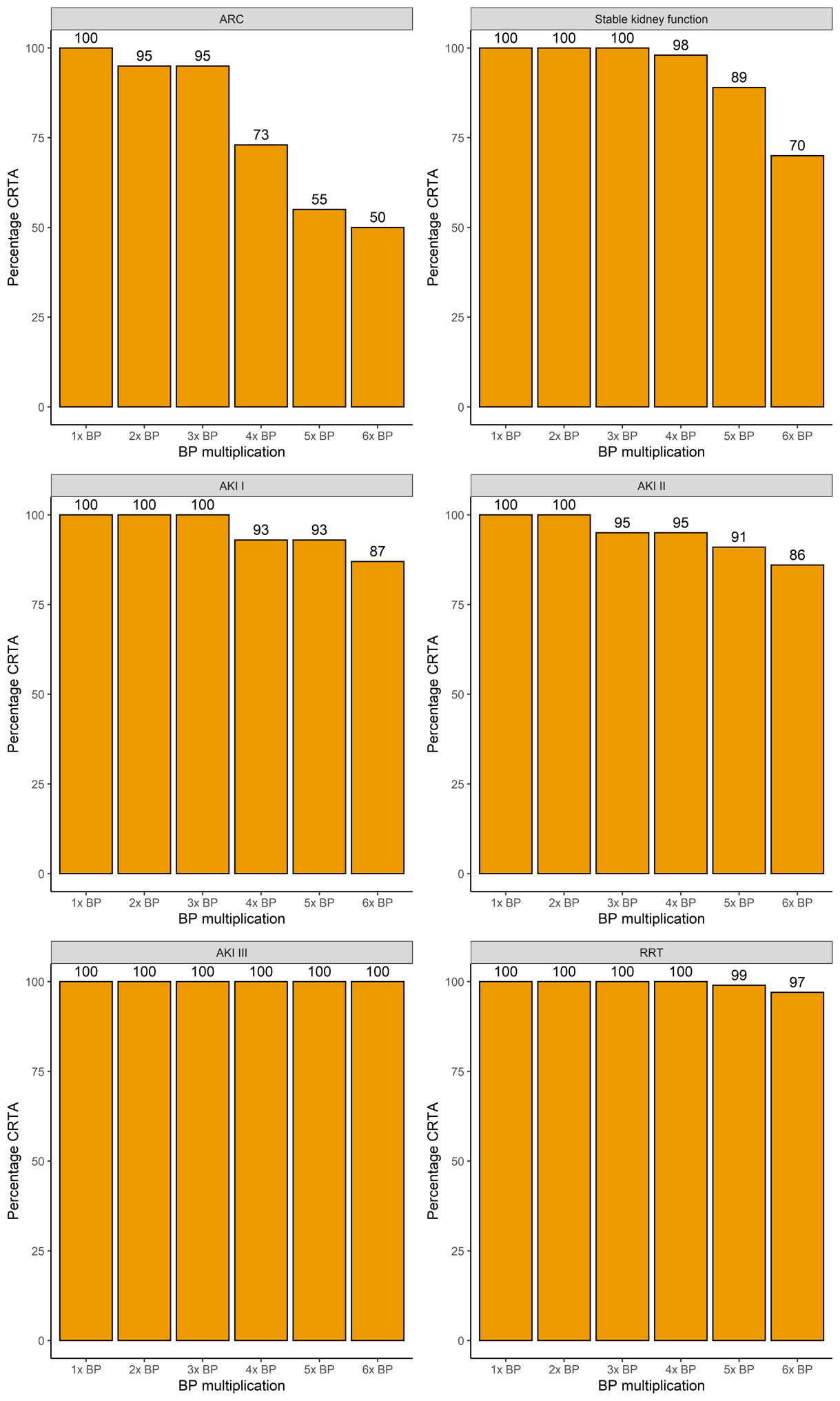


OS Figure 9 – Percentage of CRTA for MEM non-steady state samples according to renal function and BP multiplication.


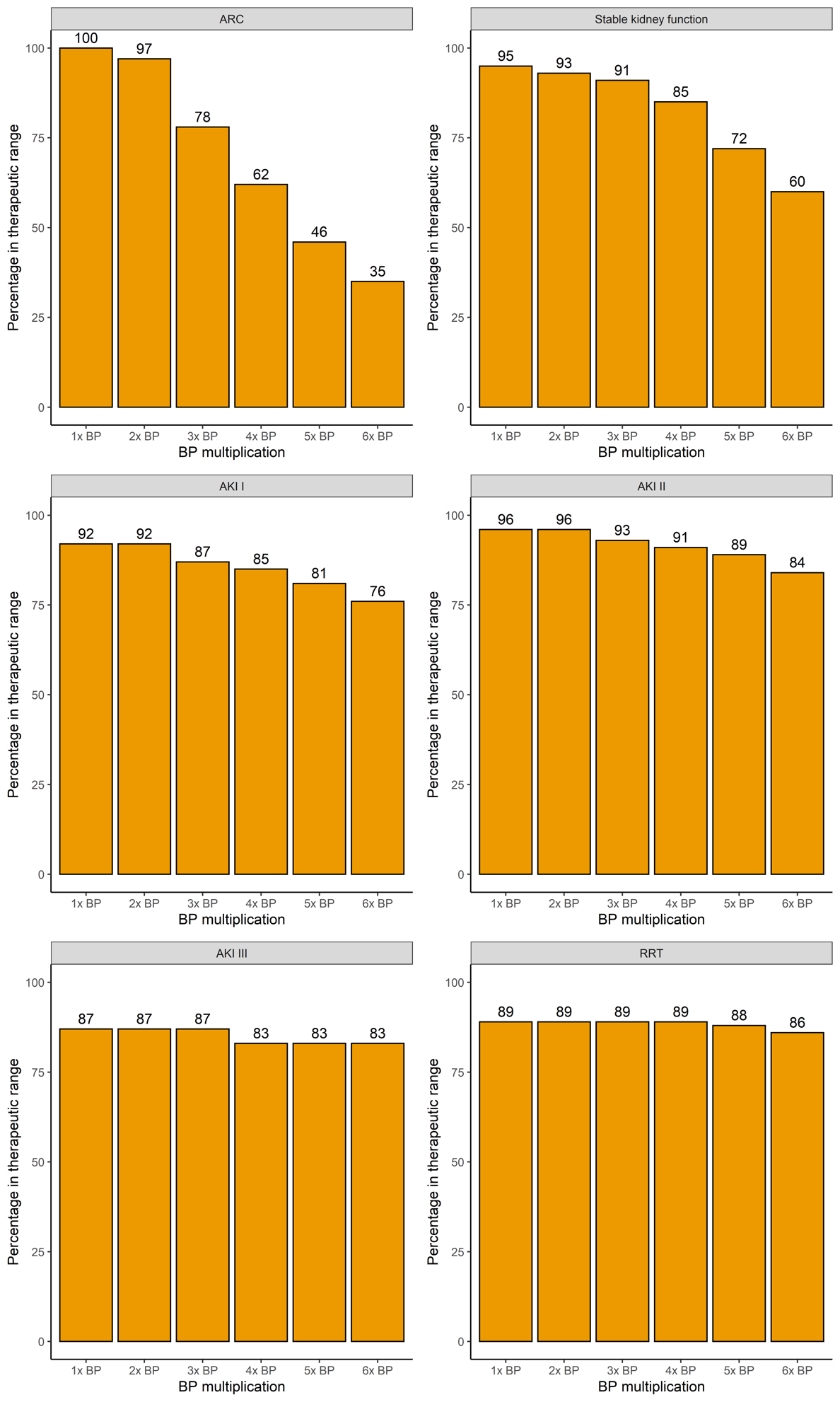


OS Figure 10: Percentage of all MEM samples within the therapeutic range for different BP multiplications according to the BP and renal function


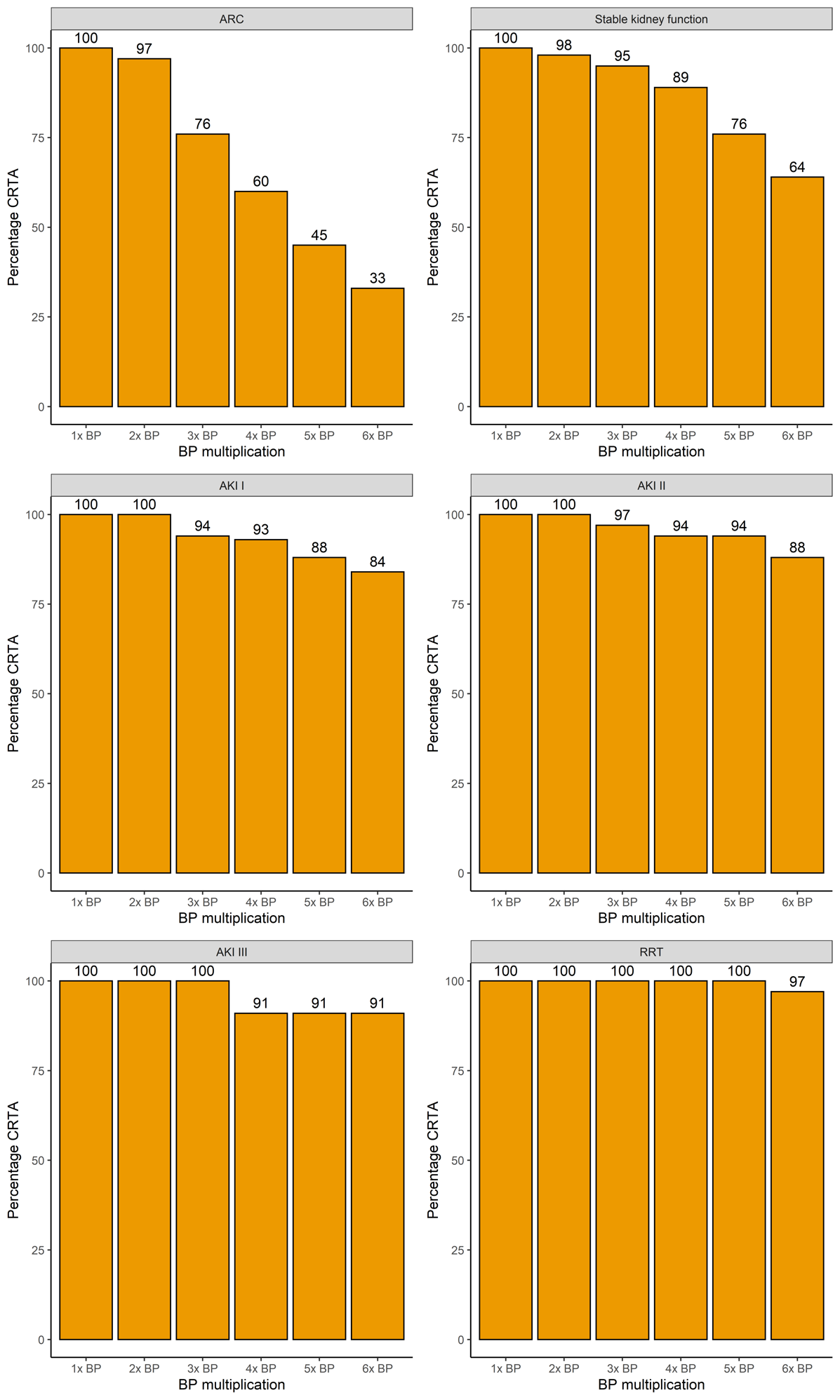


OS Figure 11: Percentage of steady state MEM samples within the therapeutic range for different BP multiplications according to the BP and renal function


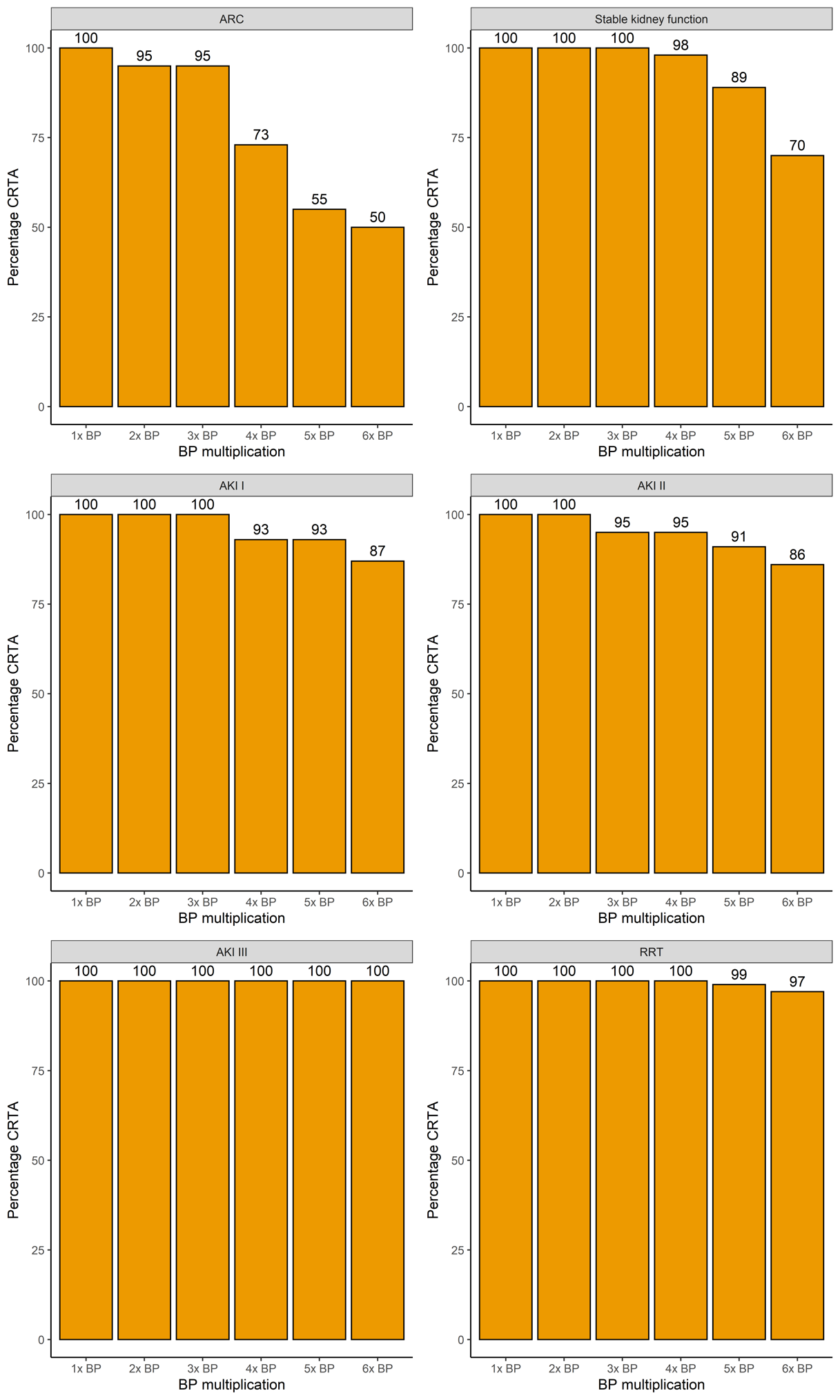


OS Figure 12: Percentage of non-steady state MEM samples within the therapeutic range for different BP multiplications according to the BP and renal function.

**EVALUATION OF MORTALITY**

When drawing up the analytical plan of the study, an effort was made to design a methodology to additionally evaluate the existence of correlation between antimicrobial concentrations/therapy adequacy and several endpoints. Unfortunately, several hurdles were identified that impeded assessing (adverse) outcomes or resolution of infection in a rigorous and scientifically valid manner. The most important one was the pragmatic nature of the study. The study approach resulted in a median number of samples per TZP treatment of 2 (IQR [1-3]) with a median time to sampling after start of the antimicrobial treatment of 2 [ 0-4] days. For MEM, this was a median of 2 [1-5] samples per treatment with a median time of sampling after start of treatment of 4 [2-7] days. There are several scenarios that can be envisioned to have occurred during one treatment period (for example: a concentration below the clinical breakpoint on day 1, above on day 2 and 3 and then again below on day 4 and 5 due to changes in renal function). As such, the number of samples per treatment available and their distribution over the treatment period is insufficient to rigorously assess the whole duration of the antimicrobial treatment. Additionally, there is the question of when an antimicrobial treatment period can be classified as “adequate” (does the concentration need to be adequate within 24h – 48h – 72h – the entire treatment period?). Furthermore, our opinion was that for such an analysis to be somewhat indicative on observational data, it would be required that patients included in this analysis were approximately at the same timepoint of their ICU admission, had approximately the same disease severity, had the same type of infection with a pathogen for which a breakpoint was available and had a sample that was drawn in steady state. Unfortunately, the patient population available did not permit us to identify a sufficiently large sub cohort.

Despite these limitations, we were intrigued by the question whether or not we could identify a similar U-shaped association between the measured concentration and mortality for TZP as was the case in the TARGET-trial (1). To this end, in a very simplistic and crude manner, we calculated the mean concentration of all TZP regimens that were prescribed for non prophylactic purposes, binned these measured concentrations in a similar manner as the TARGET-trial, and evaluated the ICU mortality rate for every bin (in the TARGET-trial, the 28-day mortality rate was compared with the measured concentration on day 1). The results of this analysis are shown in the figure below, where we compare our findings with these of the TARGET-trial. Taking into account all the caveats, interestingly enough, we found the same U-shaped association.


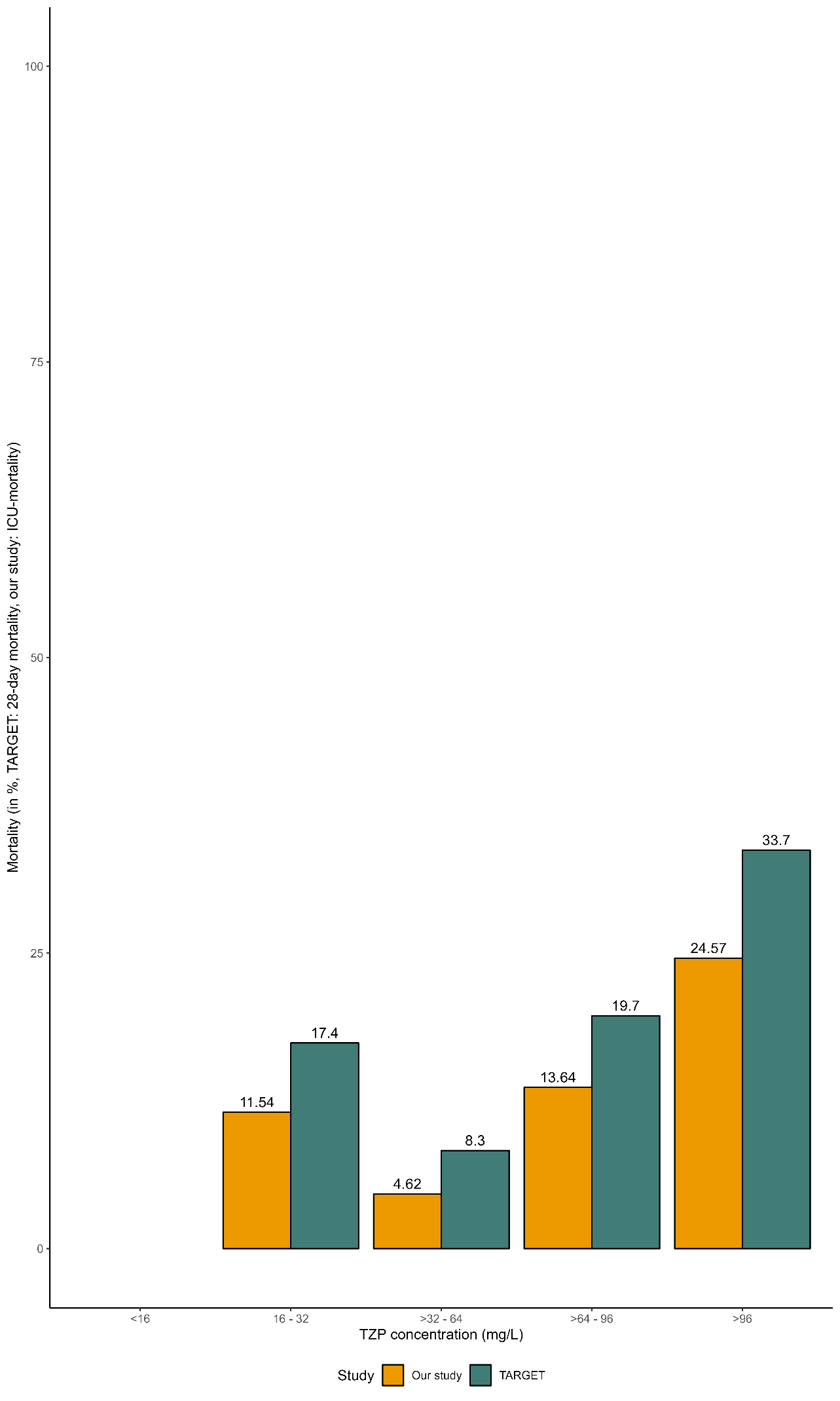


OS Figure 13: Mortality evaluation between our study and the TARGET-trial.

**REFERENCES**

1. Hagel S, Bach F, Brenner T, Bracht H, Brinkmann A, Annecke T, et al. Effect of therapeutic drug monitoring-based dose optimization of piperacillin/tazobactam on sepsis-related organ dysfunction in patients with sepsis: a randomized controlled trial. Intensive Care Med. 2022 Mar 1;48(3):311–21.
